# Syntheses, crystal structures and Hirshfeld surface analyses of four mol­ecular salts of amitriptynol

**DOI:** 10.1107/S2056989023003225

**Published:** 2023-04-14

**Authors:** Vinaya, Haleyur G. Anil Kumar, Thaluru M. Mohan Kumar, Beliyaiah Lakshmana, Yeriyur B. Basavaraju, Hemmige S. Yathirajan, Sean Parkin

**Affiliations:** a Department of Studies in Chemistry, University of Mysore, Manasagangotri, Mysuru-570 006, India; b Department of Science and Humanities, PES University, BSK III Stage, Bengaluru-560 085, India; c Department of Chemistry, Amrita School of Engineering, Amrita Vishwa Vidyapeetham, Bengaluru-560 035, India; d Department of Chemistry, University of Kentucky, Lexington, KY, 40506-0055, USA; University of Aberdeen, United Kingdom

**Keywords:** amitriptynol, amitriptyline, hydrogen bonding, Hirshfeld-surface analysis, crystal structure

## Abstract

The syntheses and crystal structures of four salts of amitriptynol (C_20_H_25_NO) with different carb­oxy­lic acids are described. The salts formed directly from solutions of amitriptyline (which first hydrolysed to amitriptynol) and the cor­responding acid in aceto­nitrile to form amitriptynolium [sys­tem­atic name: (3-{2-hy­droxy­tri­cy­clo[9.4.0.0^3,8^]penta­deca-1(11),3,5,7,12,14-hexa­en-2-yl}pro­pyl)di­methyl­az­an­ium] 4-meth­oxy­benzoate monohydrate, C_20_H_26_NO^+^·C_8_H_7_O_3_^−^·H_2_O, (**I**), ami­triptynolium 3,4-di­meth­oxy­benzoate trihydrate, C_20_H_26_NO^+^·C_9_H_9_O_4_^−^·3H_2_O, (**II**), amitriptynolium 2-chloro­benzoate, C_20_H_26_NO^+^·C_7_H_4_ClO_2_^−^, (**III**), and amitriptynolium thio­phene-2-carboxyl­ate monohydrate, C_20_H_26_NO^+^·C_5_H_3_O_2_S^−^·H_2_O, (**IV**). Compound (**III**) crystallizes with two cations, two anions and six water mol­ecules in the asymmetric unit. The different conformations of the amitriptynolium cations are determined by the torsion angles in the di­methyl­amino-propyl chains and the –CH_2_–CH_2_- bridge between the benzene rings in the tricyclic ring system, and are complicated by disorder of the bridging unit in **II** and **III**. The packing in all four salts is dominated by N—H⋯O and O—H⋯O hydrogen bonds. Hirshfeld surface analyses show that the amitriptynolium cations make similar inter-species contacts, despite the distinctly different packing in each salt.

## Abstract 2

The syntheses and low-temperature crystal structures of four organic salts of amitriptynol, a common impurity in the anti-depressant drug amitriptyline, are described.

## 1. Chemical context

Amitriptynol, C_20_H_25_NO, systematic name 5-[3-(di­methyl­amino)­prop­yl]-10,11-di­hydro-5*H*-dibenzo[*a*,*d*][7]annulen-5-ol, is a derivative and common impurity (designated ‘amitriptyline impurity B′) of amitriptyline, C_20_H_23_N. Amitriptyline is a tricyclic anti­depressant agent, which also has analgesic properties with sedative effects. Amitriptyline affects certain chemical messengers (neurotransmitters) that communicate between brain cells and help regulate mood. It is used in the treatment of depression, neuropathic pain, and migraine.

A review of the pharmacological properties and therapeutic use for chronic pain of amitriptyline was published by Bryson & Wilde (1996 ▸). A comprehensive review of amitriptyline for the treatment of fibromyalgia was given by Rico-Villademoros *et al.* (2015 ▸). In a systematic review, Thompson & Brooks (2015 ▸) discussed the use of topical amitriptyline for the treatment of neuropathic pain. A brief review of the pharmacology of amitriptyline and clinical outcomes in treating fibromyalgia was given by Lawson (2017 ▸). Analytical methods for the determination of amitriptyline and its metabolite nortriptyline were reviewed by Khatoon *et al.* (2013 ▸). Mol­ecular insights from single-crystal X-ray diffraction and DFT calculations of β-cyclo­dextrin encapsulation of nortriptyline HCl and amitriptyline HCl were published by Aree (2020*a* ▸). 
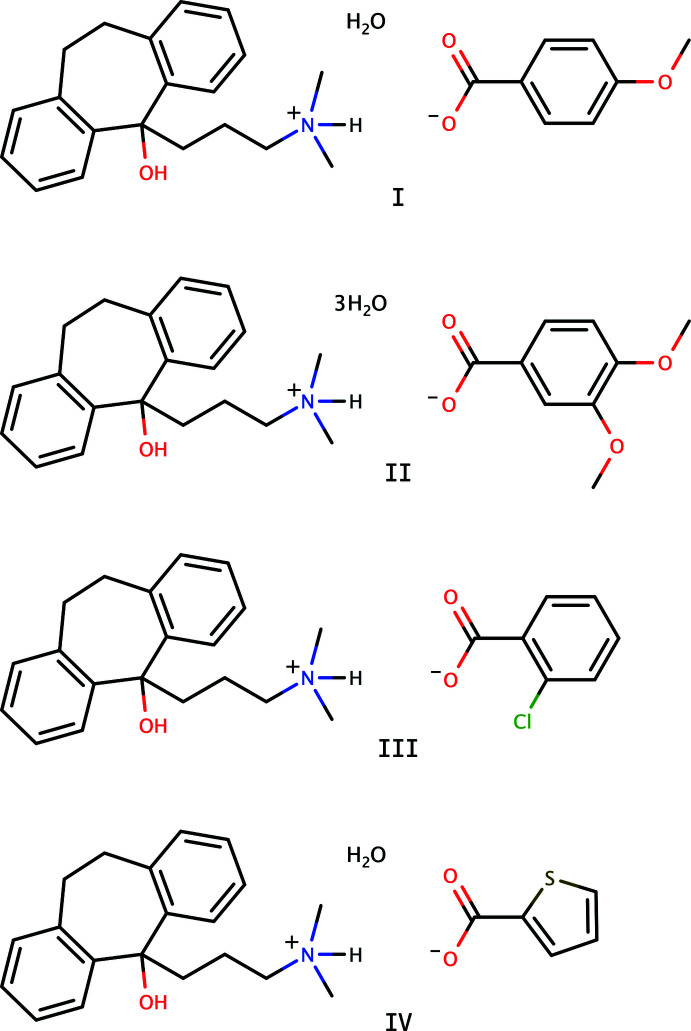


Our goal was to prepare mol­ecular salts of amitriptyline, but the amitriptyline free base is susceptible to hydrolysis, owing to its aliphatic double bond attached to the central seven-membered ring (Henwood, 1967 ▸). Consequently, the amitriptyline hydrolysed to amitriptynol, which then formed salts with the organic acids. Perhaps surprisingly, any such salts have thus far been absent from the crystallographic literature. This paper reports the crystal structures of four amitriptynolium (C_20_H_26_NO^+^) salts: 4-meth­oxy­benzoate monohydrate (**I**), 3,4-di­meth­oxy­benzoate trihydrate, (**II**), 2-chloro­benzoate (**III**) and thio­phene-2-carboxyl­ate monohydrate (**IV**).

## 2. Structural commentary

**Figure 1:**
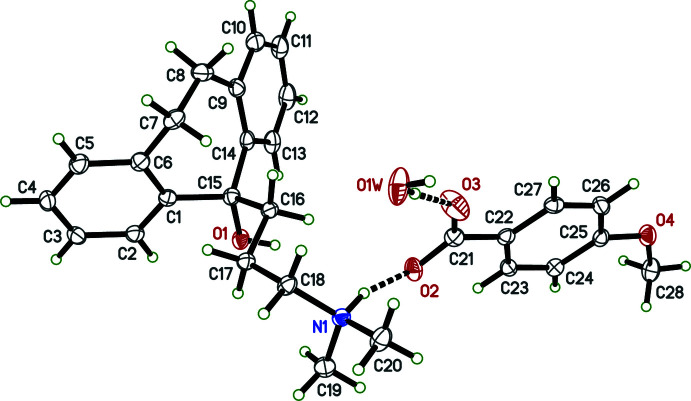
The mol­ecular structure of **I** showing 50% displacement ellipsoids. Hydrogen bonds are drawn as dashed lines.

Salts **I** and **IV** crystallized as monohydrates, and **II** as a trihydrate; only salt **III** is anhydrous (see Figs. 1 ▸–4 ▸ ▸ ▸). In spite of their chemical similarity (*i.e.*, the same cation and similar sized aromatic carboxyl­ate anions), the crystal structures of **I**–**IV** are notably distinct, each having different space-group symmetries (*Pn* for **I**, *Cc* for **II**, *P*2_1_/*n* for **III**, and *P*2_1_2_1_2_1_ for **IV**). Although only **IV** has a Sohncke space group, its structure was twinned by inversion, with major:minor twin fractions of 0.70 (7):0.30 (7), so any discussion of absolute configuration is moot.

The conformations of the amitriptynolium cations are determined by the torsion angles in the di­methyl­amino-propyl chains and by the C6—C7—C8—C9 torsion angles in the long bridge between the benzene rings of the tricyclic ring system, and are complicated by cation disorder in **II** and **III**. All conformation-defining torsion angles are given in Table 1 ▸, but further description is limited to the major disorder components. From Table 1 ▸ and Figs. 1 ▸–4 ▸ ▸ ▸, it is evident that the cation geometries in **I**, **III**, and **IV** are broadly similar. The two independent cations in **II**, however, are self-similar, but different from **I**, **III**, and **IV**, primarily evidenced by the C16—C17—C18—N1 torsion angle, which is *anti* in both cations of **II**, but *gauche* in **I**, **III**, and **IV**. In each case, the tricyclic unit of the cation adopts a ‘butterfly’ conformation with dihedral angles between the pendant benzene rings of 62.01 (9) (**I**), 69.30 (16) and 71.06 (13) (**II**), 57.21 (10) (**III**) and 50.51 (8)° (**IV**). In every case, the –OH group attached to C15 is in an equatorial orientation and the pendant alkyl chain is axial.

The 4-meth­oxy­benzoate anion in **I** is largely planar, with maximum deviation from planarity of 0.1216 (15) Å, caused by a C24—C25—O4—C28 torsion angle of −7.5 (2)° for the meth­oxy group. In **II**, the 3,4-di­meth­oxy­benzoate anions are also close to planar. In the ‘*A*’ anion, C29*A* is offset by 0.229 (2) Å from the mean plane, for a C26*A*—C25*A*—O5*A*—C29*A* meth­oxy torsion of 13.6 (3)°, while for the ‘*B*’ anion, the largest deviation is 0.2264 (16) Å for O2*B*, due to the dihedral angle between the benzene ring and the carboxyl­ate group of 10.43 (15)°. The 2-chloro­benzoate anion in **III** is disordered by a ∼180° flip, giving major:minor component occupancies of 0.9600 (15):0.0400 (15). The two components are, however, far from planar as a result of steric hindrance by the chlorine substituent; the dihedral angles between the chloro­benzene and carboxyl­ate groups being 57.82 (11)° and 56.4 (5)° for the major and minor parts, respectively. In **IV**, the thio­phene-2-carboxyl­ate anion is also disordered, with major:minor occupancies of 0.899 (3):0.101 (3), but the components are again largely planar; the maximum deviations being for O3 in each, at 0.167 (3) Å (major) and 0.14 (2) Å (minor), resulting from dihedral angles between the thio­phene rings and carboxyl­ate groups of 12.3 (6)° (major) and 11 (5)° (minor).

## 3. Supra­molecular features

The dominant supra­molecular features in all four salts are N—H⋯O hydrogen bonds between the cationic [*R*_3_N—H]^+^ moiety and the anion carboxyl­ate groups, plus O—H⋯O hydrogen bonds involving the amitriptynolium cation O—H group as donor to a carboxyl­ate acceptor in **III** and to water mol­ecules in **I**, **II**, and **IV**. These hydroxyl groups are effectively shielded from accepting strong hydrogen bonds by the adjacent benzene rings of the amitriptynolium fused ring systems in each case. The strong hydrogen bonds are augmented in all four structures by a few weaker C—H⋯O contacts.

In **I**, the main packing motifs are infinite chains of N—H⋯O and O—H⋯O hydrogen-bonded cations, anions, and water mol­ecules that extend parallel to the *a*-axis direction. These are shown in Fig. 5 ▸ and qu­anti­fied in Table 2 ▸, along with their attendant symmetry operations.

**Figure 2:**
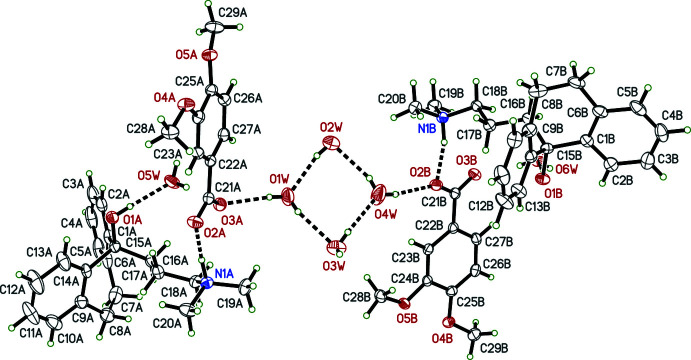
The mol­ecular structure of **II** showing 50% displacement ellipsoids. Hydrogen bonds are drawn as dashed lines. Only the major disorder component is shown.

**Figure 5:**
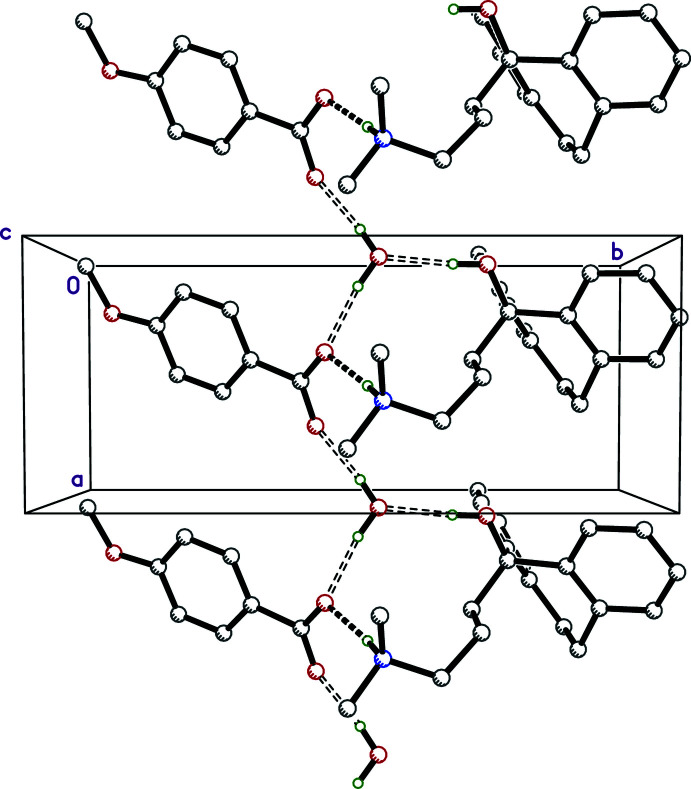
A partial packing plot of **I** viewed down the *c*-axis direction. The N—H⋯O and O—H⋯O hydrogen bonds are drawn as solid and open dashed lines, respectively. Hydrogen atoms not involved in strong hydrogen bonds are not shown.

**Figure 6:**
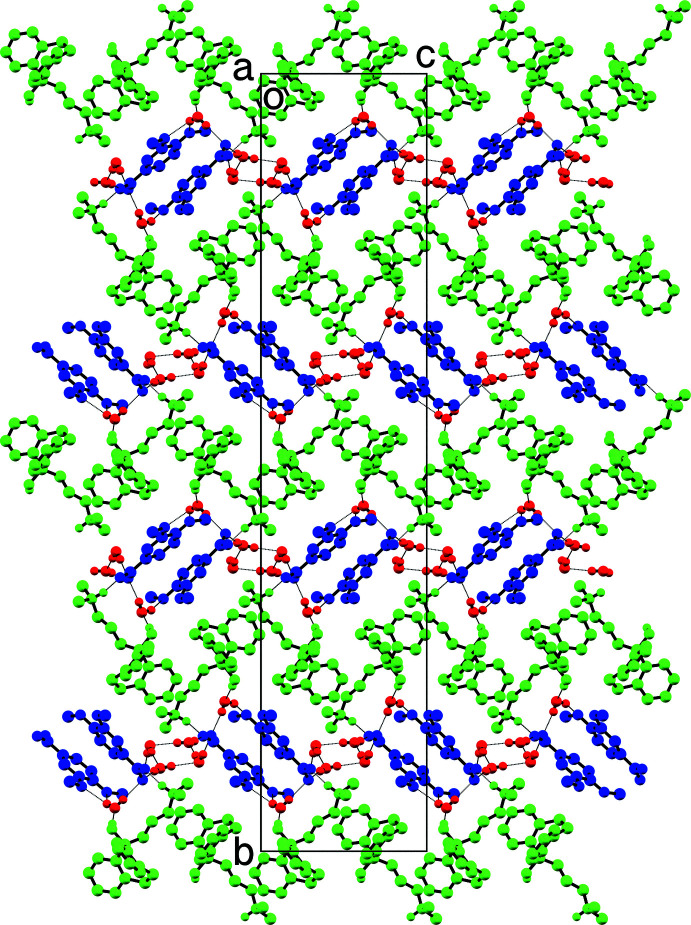
A packing plot of **II** viewed down the *a*-axis direction showing alternating layers of amitriptynolium cations (green) and 3,4-di­meth­oxy­benzoate anions (blue), inter­spersed with water mol­ecules (red). Hydrogen bonds are drawn as dotted lines.

Owing to the presence of two copies each of cation and anion, plus six water mol­ecules in the asymmetric unit (*Z*′ = 2), the packing in **II** is the most complex of the four salts. However, the most obvious supra­molecular feature, an 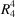 (8) ring of water mol­ecules, is evident in the ellipsoid plot of its asymmetric unit (Fig. 2 ▸). These rings of four water mol­ecules are hydrogen bonded to the anion carboxyl­ate groups (*via* O1*W* and O4*W* to O3*A* and O2*B*, respectively), and *via* O2*W* and O3*W* to (−1 + *x*, *y*, *z*) and (1 + *x*, *y*, *z*) translation-related anion carboxyl­ate groups (Table 3 ▸). The anions in turn act as hydrogen-bond acceptors to the cations (*via* O2*A* to N1*A* and O2*B* to N1*B*). The remaining water mol­ecules accept hydrogen bonds from the cation hydroxyl groups (O1*A* to O5*W* and O1*B* to O6*W*), also shown in Fig. 2 ▸. In addition to the hydrogen-bonded motifs shown in Fig. 2 ▸, water mol­ecule O5*W* takes part in bifurcated O—H⋯(O,O) hydrogen bonding to both meth­oxy groups of a translation-related (*x*, *y*, −1 + *z*) anion, and similar bifurcated hydrogen bonding occurs between water mol­ecule O6*W* and a translation-related (−1 + *x*, *y*, 1 + *z*) anion. The net result gives layers of cations and layers of anions parallel to the *ac* plane inter­spersed with and separated by the water mol­ecules (Fig. 6 ▸). These layers stack along the *b*-axis direction to build an intricate three-dimensional framework. Given its complexity and the size of the unit cell [the *b*-axis is 55.2061 (19) Å], the specific inter­actions are largely obscured, and are best viewed using a mol­ecular graphics program such as *Mercury* (Macrae *et al.*, 2020 ▸).

**Figure 7:**
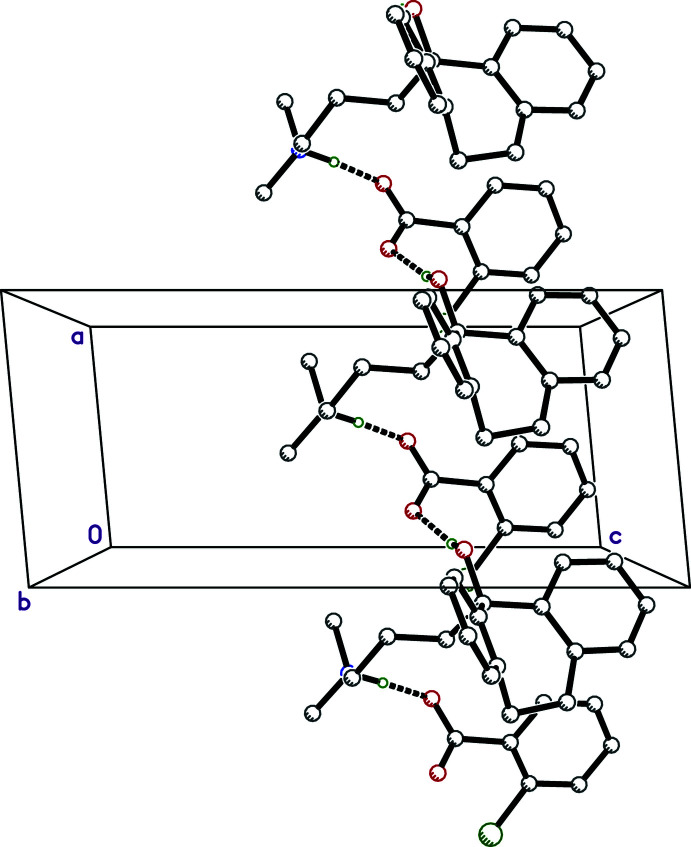
A partial packing plot of **III** viewed down the *b*-axis direction. The N—H⋯O and O—H⋯O hydrogen bonds are drawn as solid dashed lines. Minor disorder and hydrogen atoms not involved in strong hydrogen bonds are not shown.

**Figure 8:**
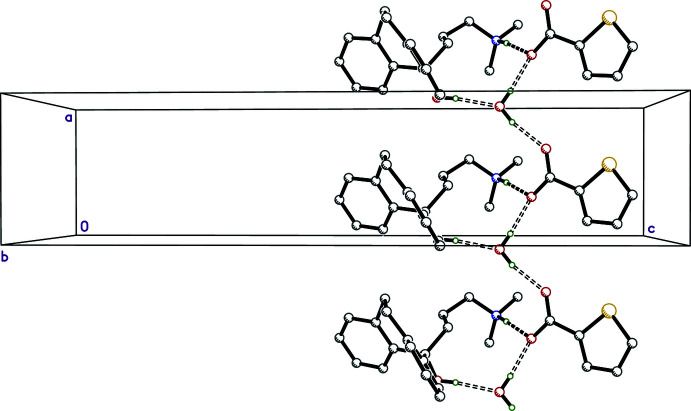
A partial packing plot of **IV** viewed down the *b*-axis direction. The N—H⋯O and O—H⋯O hydrogen bonds are drawn as solid and open dashed lines, respectively. Minor disorder and hydrogen atoms not involved in strong hydrogen bonds are not shown.

The hydrogen bonding in **III** is the simplest of the four salts because there are no water mol­ecules involved. N—H⋯O hydrogen bonds connect cation to anion within the (chosen) asymmetric unit and O—H⋯O hydrogen bonds connect cations to anions in adjacent unit cells, to form chains that extend parallel to the *a*-axis, as shown in Fig. 7 ▸ and Table 4 ▸. The main supra­molecular constructs in **IV** are hydrogen-bonded chains that propagate parallel to its *a*-axis, broadly similar to those in **I** (Fig. 8 ▸, Table 5 ▸).

**Figure 9:**
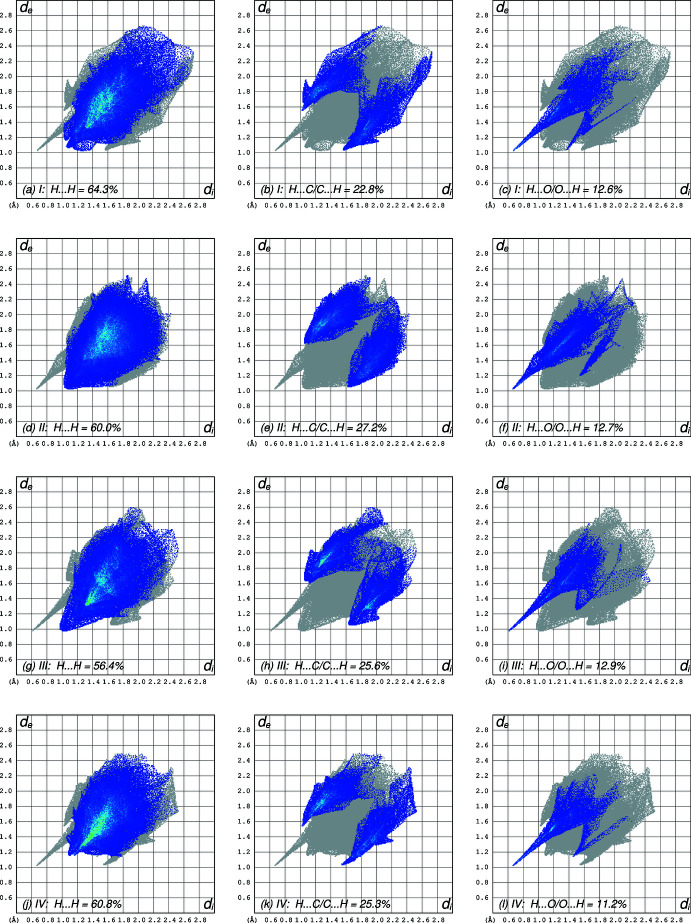
Two-dimensional Hirshfeld surface fingerprint plots showing the similarity of the main inter-species contacts for the amitriptynolium cations in: **I** [panels (*a*), (*b*), (*c*)], **II** [panels (*d*), (*e*), (*f*)], **III** [panels (*g*), (*h*), (i)], **IV** [panels (*j*), (*k*), (*l*)].

Although structures **I**–**IV** are quite different, atom-to-atom contacts involving just the amitriptynolium cations expressed in Hirshfeld-surface two-dimensional fingerprint plots (Spackman *et al.*, 2021 ▸) in each are remarkably similar, as shown in Fig. 9 ▸. The most abundant contacts are between hydrogen atoms, ranging from 56.4% in **III** to 64.3% in **I**. The next most abundant contacts are H⋯C/C⋯H, which range between 22.8% coverage in **I** to 27.2% in **II**. The only other double-digit percentage coverages are for H⋯O/O⋯H contacts, which range from 11.2% in **IV** to 12.9% in **III**. All other types of contact involving the cations are negligible.

## 4. Database survey

A search of the Cambridge Structural Database (CSD v5.43 plus updates to Nov. 2022; Groom *et al.*, 2016 ▸) for a search fragment consisting of the three fused rings with a propyl-1-amine chain attached to the seven-membered ring returned nine hits. CSD refcode CHSBHA (Wägner, 1980 ▸) has a spiro-2-cyclo­hexene-4-*N*,*N*-di­methyl­amine group in place of the propyl-1-amine chain. Entries CIKVEX and CIKVIB (Horsburgh *et al.*, 1984 ▸) are racaemic (*S*,*S* and *R*,*R*) and *meso* (*S*,*R*) penta­cyclic analogues of amitryptyline. Structures KOGXIP (Kise *et al.*, 2014 ▸), QUKDEH (Kise *et al.*, 2015 ▸), IQALUJ, IQAPEX, and IQAPIB (Kise *et al.*, 2016 ▸) carry a variety of ring-containing groups in place of the propyl-1-amine chain. Lastly, entry YEYTUS (Portalone *et al.*, 2007 ▸) is the free-base amitriptynol, from which salts **I**–**IV** were prepared. Other related structures, not returned in the above CSD search, include nortriptyline hydro­chloride (JINGIW; Klein *et al.*, 1991 ▸), three tricyclic neuroleptics (MEAPOT11, YOVYUD, and YOVZEO; Klein *et al.*, 1994 ▸), amitriptylinium picrate (DIKWEA; Bindya *et al.*, 2007 ▸), desipraminium chloride (PUKGEI; Jasinski *et al.*, 2010 ▸), desipraminium picrate (HISHEX; Swamy *et al.*, 2007 ▸), imipramine hydro­chloride and desipramine hydro­chloride (PAJTON and PALBOX; Aree, 2020*b* ▸).

## 5. Synthesis and crystallization

Solutions of commercially available (RL Fine Chem, Bengaluru, India) amitriptyline (100 mg, 0.360 mol) in methanol (10 ml) were mixed with equimolar solutions of the appropriate acid in aceto­nitrile (10 ml) *viz*., 4-meth­oxy­benzoic acid (55 mg, 0.360 mol) for **I**, 3,4-di­meth­oxy­benzoic acid (67 mg, 0.483 mol) for **II**, 2-chloro­benzoic acid (57 mg, 0.360 mol) for **III** and thio­phene 2-carb­oxy­lic acid (46 mg, 0.360 mol) for **IV**. The resulting solutions were stirred for 30 minutes at 333 K and allowed to stand at room temperature. X-ray quality crystals formed on slow evaporation of solutions in ethanol:aceto­nitrile (1:1) after a week for all four compounds. The melting points are 367–369 K (**I**), 359–361 K (**II**), 410–412 K (**III**) and 373–376 K (**IV**).

## 6. Refinement

Crystal data, data collection, and refinement statistics are given in Table 6 ▸. Crystals of **III** shattered on cooling to 90 K, but remained intact at 180 K. Non-disordered hydrogen atoms were located in difference-Fourier maps. Those bound to nitro­gen or oxygen atoms were refined, but carbon-bound hydrogen atoms were included using riding models with constrained distances of 0.95 Å (C*sp* ^2^H), 0.99 Å (*R*_2_CH_2_), and 0.98 Å (*R*CH_3_) using *U*_iso_(H) values constrained to 1.2*U*_eq_ or 1.5*U*_eq_ (methyl group only) of the attached carbon atom. Structure **IV** was twinned by inversion, which was included using the standard TWIN/BASF treatment in *SHELXL*. Two-component disorder in the amitriptynolium cations of **II** and **III** and the anions of **III** and **IV** was handled using separate PART instructions and occupancies set *via* FVAR parameters in *SHELXL*.

## Supplementary Material

Crystal structure: contains datablock(s) I, II, III, IV, global. DOI: 10.1107/S2056989023003225/hb8061sup1.cif

Structure factors: contains datablock(s) I. DOI: 10.1107/S2056989023003225/hb8061Isup2.hkl

Structure factors: contains datablock(s) II. DOI: 10.1107/S2056989023003225/hb8061IIsup3.hkl

Structure factors: contains datablock(s) III. DOI: 10.1107/S2056989023003225/hb8061IIIsup4.hkl

Structure factors: contains datablock(s) IV. DOI: 10.1107/S2056989023003225/hb8061IVsup5.hkl

Supporting information file. DOI: 10.1107/S2056989023003225/hb8061Isup6.cml

Supporting information file. DOI: 10.1107/S2056989023003225/hb8061IIIsup7.cml

Supporting information file. DOI: 10.1107/S2056989023003225/hb8061IVsup8.cml

CCDC references: 2254696, 2254695, 2254694, 2254693

Additional supporting information:  crystallographic information; 3D view; checkCIF report

## Figures and Tables

**Figure 3:**
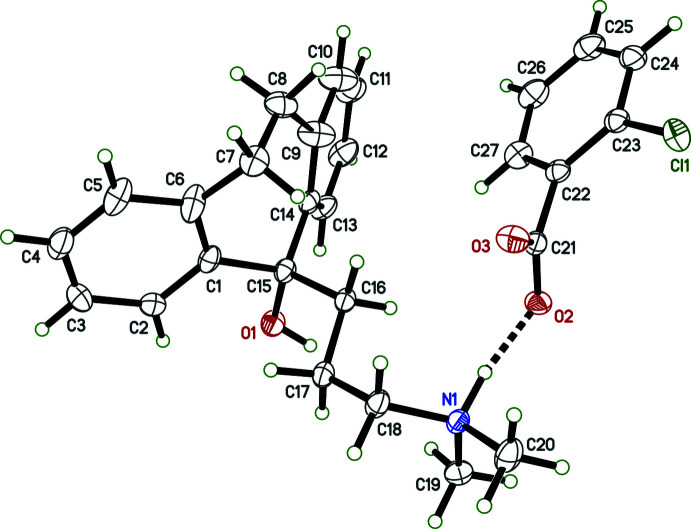
The mol­ecular structure of **III** showing 50% displacement ellipsoids. Hydrogen bonds are drawn as dashed lines. Only the major disorder component is shown.

**Figure 4:**
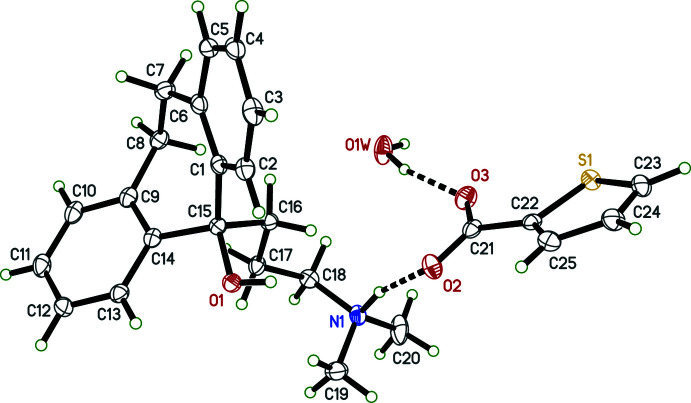
The mol­ecular structure of **IV** showing 50% displacement ellipsoids. Hydrogen bonds are drawn as dashed lines. Only the major disorder component is shown.

**Table 1:** Conformation-defining torsion angles (°) in **I**–**IV**

atoms	torsion angle	geometry
**I**		
C6—C7—C8—C9	−50.9 (2)	*gauche*
C15—C16—C17—C18	172.16 (14)	*anti*
C16—C17—C18—N1	−71.7 (2)	*gauche*
**II**		
C6*A*—C7*A*—C8*A*—C9*A*	18.7 (12)	*syn*
C6*A*—C7*A*′—C8*A*′—C9*A*	−38 (3)	*gauche*
C6*B*—C7*B*—C8*B*—C9*B*	17.7 (9)	*syn*
C6*B*—C7*B*′—C8*B*′—C9*B*	−35 (3)	*gauche*
C15*A*—C16*A*—C17*A*—C18*A*	−172.24 (19)	*anti*
C16*A*—C17*A*—C18*A*—N1*A*	162.68 (19)	*anti*
C15*B*—C16*B*—C17*B*—C18*B*	−156.6 (2)	*anti*
C16*B*—C17*B*—C18*B*—N1*B*	167.64 (19)	*anti*
**III**		
C6—C7—C8—C9	−64.1 (4)	*gauche*
C6—C7′—C8′—C9	69.8 (5)	*gauche*
C15—C16—C17—C18	−168.84 (14)	*anti*
C16—C17—C18—N1	−64.1 (2)	*gauche*
**IV**		
C6—C7—C8—C9	56.9 (2)	*gauche*
C15—C16—C17—C18	−160.52 (15)	*anti*
C16—C17—C18—N1	68.6 (2)	*gauche*

**Table 2:** Hydrogen-bond geometry (Å, °) for **I** [Chem scheme1]

*D*—H⋯*A*	*D*—H	H⋯*A*	*D*⋯*A*	*D*—H⋯*A*
O1—H1*O*⋯O1*W* ^i^	0.85 (3)	1.84 (3)	2.6911 (18)	171 (3)
N1—H1*N*⋯O2	0.94 (2)	1.76 (2)	2.6711 (18)	163 (2)
C18—H18*A*⋯O1^ii^	0.99	2.50	3.400 (2)	151
C18—H18*A*⋯O1*W*	0.99	2.66	3.523 (3)	146
C19—H19*C*⋯O3^iii^	0.98	2.59	3.191 (3)	119
C20—H20*C*⋯O1*W*	0.98	2.54	3.420 (3)	149
O1*W*—H1*W*1⋯O3	0.84 (3)	1.81 (3)	2.646 (2)	173 (3)
O1*W*—H2*W*1⋯O2^ii^	0.90 (3)	1.84 (3)	2.718 (2)	165 (3)

Symmetry codes: (i) 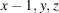 ; (ii) 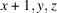 ; (iii) 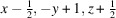 .

**Table 3:** Hydrogen-bond geometry (Å, °) for **II** [Chem scheme1]

*D*—H⋯*A*	*D*—H	H⋯*A*	*D*⋯*A*	*D*—H⋯*A*
O1*A*—H1*OA*⋯O5*W*	0.82 (4)	1.95 (4)	2.762 (3)	172 (4)
N1*A*—H1*NA*⋯O2*A*	0.99 (3)	1.71 (3)	2.690 (3)	172 (3)
N1*A*—H1*NA*⋯O3*A*	0.99 (3)	2.45 (3)	3.108 (3)	124 (2)
C16*A*—H16*B*⋯O5*W*	0.99	2.61	3.308 (3)	128
C23*A*—H23*A*⋯O2*W* ^i^	0.95	2.61	3.519 (3)	159
O1*B*—H1*OB*⋯O6*W*	0.87 (4)	1.91 (4)	2.781 (3)	173 (3)
N1*B*—H1*NB*⋯O2*B*	0.99 (3)	1.75 (3)	2.723 (3)	165 (2)
N1*B*—H1*NB*⋯O3*B*	0.99 (3)	2.48 (3)	3.186 (3)	128 (2)
C16*B*—H16*D*⋯O6*W*	0.99	2.46	3.134 (3)	125
O1*W*—H1*W*1⋯O3*A*	0.82 (2)	1.96 (2)	2.770 (3)	172 (4)
O1*W*—H2*W*1⋯O3*W*	0.81 (2)	2.02 (2)	2.795 (3)	161 (4)
O2*W*—H1*W*2⋯O2*A* ^ii^	0.83 (2)	1.95 (2)	2.773 (3)	170 (4)
O2*W*—H2*W*2⋯O1*W*	0.83 (2)	1.93 (2)	2.738 (3)	166 (4)
O3*W*—H1*W*3⋯O3*B* ^i^	0.83 (2)	1.96 (2)	2.785 (3)	178 (5)
O3*W*—H2*W*3⋯O4*W*	0.83 (2)	1.88 (2)	2.705 (4)	174 (5)
O4*W*—H1*W*4⋯O2*B*	0.82 (2)	1.89 (2)	2.708 (3)	171 (4)
O4*W*—H2*W*4⋯O2*W*	0.81 (2)	1.99 (2)	2.771 (3)	160 (4)
O5*W*—H1*W*5⋯O4*B* ^iii^	0.83 (2)	2.36 (3)	3.035 (3)	138 (3)
O5*W*—H1*W*5⋯O5*B* ^iii^	0.83 (2)	2.23 (2)	2.971 (2)	149 (3)
O5*W*—H2*W*5⋯O3*A*	0.83 (2)	2.00 (2)	2.820 (3)	173 (4)
O6*W*—H1*W*6⋯O4*A* ^iv^	0.84 (2)	2.24 (3)	2.886 (2)	134 (3)
O6*W*—H1*W*6⋯O5*A* ^iv^	0.84 (2)	2.23 (2)	2.980 (3)	150 (3)
O6*W*—H2*W*6⋯O3*B*	0.83 (2)	1.95 (2)	2.769 (3)	169 (3)

Symmetry codes: (i) 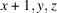 ; (ii) 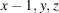 ; (iii) 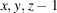 ; (iv) 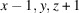 .

**Table 4:** Hydrogen-bond geometry (Å, °) for **III** [Chem scheme1]

*D*—H⋯*A*	*D*—H	H⋯*A*	*D*⋯*A*	*D*—H⋯*A*
O1—H1*O*⋯O3^i^	0.86 (2)	1.91 (2)	2.724 (3)	159 (2)
O1—H1*O*⋯O3′^i^	0.86 (2)	2.02 (8)	2.81 (8)	153 (3)
N1—H1*N*⋯O2	1.00 (2)	1.61 (2)	2.605 (2)	171.5 (19)
N1—H1*N*⋯O2′	1.00 (2)	1.73 (4)	2.73 (4)	178 (4)
N1—H1*N*⋯O3′	1.00 (2)	2.58 (5)	3.23 (5)	122.4 (16)
C19—H19*B*⋯O3^i^	0.98	2.63	3.595 (4)	167
C19—H19*B*⋯O3′^i^	0.98	2.52	3.47 (9)	162
C20—H20*C*⋯O2^ii^	0.98	2.46	3.426 (4)	169
C20—H20*C*⋯O2′^ii^	0.98	2.57	3.54 (10)	169

Symmetry codes: (i) 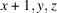 ; (ii) 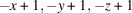 .

**Table 5:** Hydrogen-bond geometry (Å, °) for **IV** [Chem scheme1]

*D*—H⋯*A*	*D*—H	H⋯*A*	*D*⋯*A*	*D*—H⋯*A*
O1—H1*O*⋯O1*W* ^i^	0.82 (2)	1.95 (2)	2.7607 (18)	170 (2)
N1—H1*N*⋯O2	0.95 (2)	1.74 (2)	2.664 (2)	164 (2)
C18—H18*A*⋯S1′b^ii^	0.99	2.93	3.90 (3)	167
C18—H18*B*⋯O1*W*	0.99	2.53	3.495 (3)	164
C19—H19*C*⋯O3^ii^	0.98	2.46	3.388 (3)	158
C25′b—H25′b⋯S1′b^iii^	0.95	2.90	3.82 (3)	165
O1*W*—H1*W*⋯O3	0.87 (3)	1.85 (3)	2.6993 (19)	165 (3)
O1*W*—H2*W*⋯O2^iii^	0.83 (3)	1.89 (3)	2.716 (2)	174 (3)

Symmetry codes: (i) 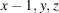 ; (ii) 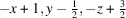 ; (iii) 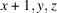 .

**Table 6:** Experimental details

	**I**	**II**	**III**	**IV**
Crystal data				
Chemical formula	C_20_H_26_NO^+^·C_8_H_7_O_3_^−^·H_2_O	C_20_H_26_NO^+^·C_9_H_9_O_4_^−^·3H_2_O	C_20_H_26_NO^+^·C_7_H_4_ClO_2_^−^	C_20_H_26_NO^+^·C_5_H_3_O_2_S^−^·H_2_O
*M*_r_	465.57	531.63	451.97	441.57
Crystal system, space group	Monoclinic, *P* *n*	Monoclinic, *C* *c*	Monoclinic, *P*2_1_/*n*	Orthorhombic, *P*2_1_2_1_2_1_
Temperature (K)	90	90	180	90
*a*, *b*, *c* (Å)	6.2398 (2), 14.7216 (4), 13.5383 (4)	8.6750 (3), 55.2061 (19), 12.3988 (4)	6.7576 (2), 22.9081 (6), 14.9477 (3)	6.1659 (5), 13.1299 (12), 27.698 (2)
α, β, γ (°)	90, 94.229 (1), 90	90, 108.238 (2), 90	90, 95.359 (1), 90	90, 90, 90
*V* (Å^3^)	1240.24 (6)	5639.7 (3)	2303.85 (10)	2242.3 (3)
*Z*	2	8	4	4
Radiation type	Cu *K*α	Cu *K*α	Mo *K*α	Mo *K*α
μ (mm^−1^)	0.68	0.74	0.20	0.18
Crystal size (mm)	0.30 × 0.24 × 0.18	0.30 × 0.24 × 0.18	0.22 × 0.16 × 0.12	0.27 × 0.13 × 0.04

Data collection				
Diffractometer	Bruker D8 Venture dual source	Bruker D8 Venture dual source	Bruker D8 Venture dual source	Bruker D8 Venture dual source
Absorption correction	Multi-scan (*SADABS*; Krause *et al.*, 2015 ▸)	Multi-scan (*SADABS*; Krause *et al.*, 2015 ▸)	Multi-scan (*SADABS*; Krause *et al.*, 2015 ▸)	Multi-scan (*SADABS*; Krause *et al.*, 2015 ▸)
*T*_min_, *T*_max_	0.893, 0.971	0.858, 0.982	0.848, 0.959	0.852, 0.959
No. of measured, independent and observed [*I* > 2σ(*I*)] reflections	15326, 4078, 3995	22593, 9069, 8333	35872, 5279, 4188	43123, 5133, 4772
*R*_int_	0.022	0.034	0.039	0.042
(sin θ/λ)_max_ (Å^−1^)	0.625	0.625	0.650	0.650

Refinement				
*R*[*F* ^2^ > 2σ(*F* ^2^)], *wR*(*F* ^2^), *S*	0.024, 0.064, 1.02	0.031, 0.074, 1.02	0.045, 0.110, 1.03	0.028, 0.062, 1.06
No. of reflections	4078	9069	5279	5133
No. of parameters	327	773	380	315
No. of restraints	3	160	404	10
H-atom treatment	H atoms treated by a mixture of independent and constrained refinement	H atoms treated by a mixture of independent and constrained refinement	H atoms treated by a mixture of independent and constrained refinement	H atoms treated by a mixture of independent and constrained refinement
Δρ_max_, Δρ_min_ (e Å^−3^)	0.16, −0.14	0.17, −0.20	0.63, −0.61	0.19, −0.19
Absolute structure	Flack *x* obtained from 1479 quotients [(*I* ^+^)−(*I* ^−^)]/[(*I* ^+^)+(*I* ^−^)] (Parsons *et al.*, 2013 ▸)	Flack *x* obtained from 2935 quotients [(*I* ^+^)−(*I* ^−^)]/[(*I* ^+^)+(*I* ^−^)] (Parsons *et al.*, 2013 ▸)	–	Twinned by inversion
Absolute structure parameter	0.08 (7)	0.00 (7)	–	0.30 (7)

Computer programs: *APEX3* (Bruker, 2016 ▸), *SHELXT* (Sheldrick, 2015*a* ▸), *SHELXL2019/2* (Sheldrick, 2015*b* ▸), *XP* in *SHELXTL* (Sheldrick, 2008 ▸), *Mercury* (Macrae *et al.*, 2020 ▸), *SHELX* (Sheldrick, 2008 ▸) and *publCIF* (Westrip, 2010 ▸).

## supplementary crystallographic information

### (3-{2-Hydroxytricyclo[9.4.0.03,8]pentadeca-1(11),3,5,7,12,14-hexaen-2-yl}propyl)dimethylazanium 4-methoxybenzoate monohydrate (I) Crystal data

**Table:**

C_20_H_26_NO^+^·C_8_H_7_O_3_^−^·H_2_O	*F*(000) = 500
*M**_r_* = 465.57	*D*_x_ = 1.247 Mg m^−^^3^
Monoclinic, *P**n*	Cu *K*α radiation, λ = 1.54178 Å
*a* = 6.2398 (2) Å	Cell parameters from 9880 reflections
*b* = 14.7216 (4) Å	θ = 3.0–74.4°
*c* = 13.5383 (4) Å	µ = 0.68 mm^−^^1^
β = 94.229 (1)°	*T* = 90 K
*V* = 1240.24 (6) Å^3^	Cut block, colourless
*Z* = 2	0.30 × 0.24 × 0.18 mm

### (3-{2-Hydroxytricyclo[9.4.0.03,8]pentadeca-1(11),3,5,7,12,14-hexaen-2-yl}propyl)dimethylazanium 4-methoxybenzoate monohydrate (I) Data collection

**Table:**

Bruker D8 Venture dual source diffractometer	4078 independent reflections
Radiation source: microsource	3995 reflections with *I* > 2σ(*I*)
Detector resolution: 7.41 pixels mm^-1^	*R*_int_ = 0.022
φ and ω scans	θ_max_ = 74.4°, θ_min_ = 3.0°
Absorption correction: multi-scan (*SADABS*; Krause *et al.*, 2015)	*h* = −6→7
*T*_min_ = 0.893, *T*_max_ = 0.971	*k* = −18→18
15326 measured reflections	*l* = −16→16

### (3-{2-Hydroxytricyclo[9.4.0.03,8]pentadeca-1(11),3,5,7,12,14-hexaen-2-yl}propyl)dimethylazanium 4-methoxybenzoate monohydrate (I) Refinement

**Table:**

Refinement on *F*^2^	Hydrogen site location: mixed
Least-squares matrix: full	H atoms treated by a mixture of independent and constrained refinement
*R*[*F*^2^ > 2σ(*F*^2^)] = 0.024	*w* = 1/[σ^2^(*F*_o_^2^) + (0.032*P*)^2^ + 0.2099*P*] where *P* = (*F*_o_^2^ + 2*F*_c_^2^)/3
*wR*(*F*^2^) = 0.064	(Δ/σ)_max_ = 0.001
*S* = 1.02	Δρ_max_ = 0.16 e Å^−^^3^
4078 reflections	Δρ_min_ = −0.14 e Å^−^^3^
327 parameters	Extinction correction: *SHELXL2019/2* (Sheldrick, 2015b), Fc^*^=kFc[1+0.001xFc^2^λ^3^/sin(2θ)]^-1/4^
3 restraints	Extinction coefficient: 0.0035 (6)
Primary atom site location: structure-invariant direct methods	Absolute structure: Flack *x* obtained from 1479 quotients [(*I*^+^)-(*I*^-^)]/[(*I*^+^)+(*I*^-^)] (Parsons *et al.*, 2013)
Secondary atom site location: difference Fourier map	Absolute structure parameter: 0.08 (7)

### (3-{2-Hydroxytricyclo[9.4.0.03,8]pentadeca-1(11),3,5,7,12,14-hexaen-2-yl}propyl)dimethylazanium 4-methoxybenzoate monohydrate (I) Special details

**Table:**

Experimental. The crystal was mounted using polyisobutene oil on the tip of a fine glass fibre, which was fastened in a copper mounting pin with electrical solder. It was placed directly into the cold gas stream of a liquid-nitrogen based cryostat (Hope, 1994; Parkin & Hope, 1998). Diffraction data were collected with the crystal at 90K, which is standard practice in this laboratory for the majority of flash-cooled crystals.
Geometry. All esds (except the esd in the dihedral angle between two l.s. planes) are estimated using the full covariance matrix. The cell esds are taken into account individually in the estimation of esds in distances, angles and torsion angles; correlations between esds in cell parameters are only used when they are defined by crystal symmetry. An approximate (isotropic) treatment of cell esds is used for estimating esds involving l.s. planes.
Refinement. Refinement progress was checked using *Platon* (Spek, 2020) and by an *R*-tensor (Parkin, 2000). The final model was further checked with the IUCr utility *checkCIF*.

### (3-{2-Hydroxytricyclo[9.4.0.03,8]pentadeca-1(11),3,5,7,12,14-hexaen-2-yl}propyl)dimethylazanium 4-methoxybenzoate monohydrate (I) Fractional atomic coordinates and isotropic or equivalent isotropic displacement parameters (Å^2^)

**Table:**

	*x*	*y*	*z*	*U*_iso_*/*U*_eq_	
O1	0.05552 (19)	0.72439 (8)	0.57107 (10)	0.0207 (3)	
H1O	0.053 (4)	0.6672 (19)	0.561 (2)	0.039 (7)*	
N1	0.5936 (2)	0.54909 (9)	0.72662 (11)	0.0169 (3)	
H1N	0.537 (3)	0.5239 (15)	0.6666 (18)	0.021 (5)*	
C1	0.2571 (3)	0.85982 (11)	0.57192 (12)	0.0169 (3)	
C2	0.0971 (3)	0.89257 (12)	0.62852 (13)	0.0195 (3)	
H2	−0.017827	0.853518	0.642767	0.023*	
C3	0.1005 (3)	0.98071 (12)	0.66482 (13)	0.0224 (4)	
H3	−0.012447	1.001724	0.702307	0.027*	
C4	0.2695 (3)	1.03815 (12)	0.64621 (13)	0.0240 (4)	
H4	0.273384	1.098709	0.670526	0.029*	
C5	0.4316 (3)	1.00568 (12)	0.59177 (14)	0.0231 (4)	
H5	0.548457	1.044559	0.579907	0.028*	
C6	0.4294 (3)	0.91754 (12)	0.55353 (13)	0.0197 (3)	
C7	0.6095 (3)	0.88649 (12)	0.49423 (14)	0.0220 (4)	
H7A	0.670947	0.829778	0.523644	0.026*	
H7B	0.724160	0.933119	0.498402	0.026*	
C8	0.5390 (3)	0.86947 (13)	0.38562 (14)	0.0242 (4)	
H8A	0.511844	0.929638	0.354531	0.029*	
H8B	0.663866	0.843058	0.354795	0.029*	
C9	0.3461 (3)	0.81008 (11)	0.35504 (13)	0.0207 (4)	
C10	0.3015 (4)	0.80517 (12)	0.25206 (14)	0.0279 (4)	
H10	0.399634	0.832237	0.210388	0.033*	
C11	0.1211 (4)	0.76264 (12)	0.20880 (14)	0.0318 (5)	
H11	0.095222	0.761135	0.138778	0.038*	
C12	−0.0207 (3)	0.72250 (12)	0.26846 (16)	0.0299 (5)	
H12	−0.147085	0.693966	0.239991	0.036*	
C13	0.0220 (3)	0.72390 (11)	0.37074 (15)	0.0238 (4)	
H13	−0.076086	0.695359	0.411320	0.029*	
C14	0.2061 (3)	0.7663 (1)	0.41590 (13)	0.0181 (3)	
C15	0.2394 (3)	0.76263 (11)	0.53007 (13)	0.0170 (3)	
C16	0.4325 (3)	0.70040 (11)	0.56309 (12)	0.0165 (3)	
H16B	0.391728	0.636220	0.550513	0.020*	
H16A	0.554670	0.714706	0.523040	0.020*	
C17	0.5035 (3)	0.71235 (11)	0.67311 (12)	0.0196 (4)	
H17A	0.561491	0.774523	0.683229	0.024*	
H17B	0.375332	0.707074	0.711697	0.024*	
C18	0.6713 (3)	0.64510 (11)	0.71430 (13)	0.0197 (3)	
H18A	0.790325	0.644083	0.669854	0.024*	
H18B	0.730746	0.667431	0.779651	0.024*	
C19	0.4136 (3)	0.54363 (13)	0.79194 (15)	0.0274 (4)	
H19A	0.376582	0.479798	0.802175	0.041*	
H19B	0.288444	0.575669	0.760923	0.041*	
H19C	0.456773	0.571815	0.855914	0.041*	
C20	0.7769 (3)	0.49130 (12)	0.76608 (15)	0.0259 (4)	
H20A	0.728457	0.428261	0.771203	0.039*	
H20B	0.829547	0.513304	0.831755	0.039*	
H20C	0.892900	0.494386	0.721206	0.039*	
O2	0.4034 (2)	0.45194 (8)	0.5783 (1)	0.0234 (3)	
O3	0.6984 (2)	0.43518 (11)	0.49734 (13)	0.0426 (4)	
O4	0.2349 (2)	0.07544 (8)	0.35022 (10)	0.0245 (3)	
C21	0.5175 (3)	0.41034 (11)	0.51922 (13)	0.0198 (3)	
C22	0.4306 (3)	0.32348 (11)	0.47354 (12)	0.0169 (3)	
C23	0.2284 (3)	0.28982 (11)	0.49120 (12)	0.0180 (3)	
H23	0.138870	0.323687	0.531451	0.022*	
C24	0.1549 (3)	0.20751 (11)	0.45102 (13)	0.0186 (3)	
H24	0.015986	0.185752	0.463216	0.022*	
C25	0.2868 (3)	0.15731 (11)	0.39276 (13)	0.0184 (3)	
C26	0.4889 (3)	0.19091 (11)	0.37383 (13)	0.0204 (4)	
H26	0.578018	0.157350	0.333126	0.024*	
C27	0.5595 (3)	0.27239 (11)	0.41385 (13)	0.0189 (3)	
H27	0.697803	0.294343	0.400821	0.023*	
C28	0.0414 (3)	0.03267 (12)	0.37610 (16)	0.0271 (4)	
H28A	0.028544	−0.027200	0.344564	0.041*	
H28B	−0.082092	0.070258	0.353305	0.041*	
H28C	0.045039	0.025594	0.448178	0.041*	
O1W	1.0235 (3)	0.54257 (9)	0.55570 (14)	0.0366 (4)	
H1W1	0.913 (5)	0.511 (2)	0.539 (2)	0.052 (8)*	
H2W1	1.141 (5)	0.508 (2)	0.553 (2)	0.051 (8)*	

### (3-{2-Hydroxytricyclo[9.4.0.03,8]pentadeca-1(11),3,5,7,12,14-hexaen-2-yl}propyl)dimethylazanium 4-methoxybenzoate monohydrate (I) Atomic displacement parameters (Å^2^)

**Table:**

	*U*^11^	*U*^22^	*U*^33^	*U*^12^	*U*^13^	*U*^23^
O1	0.0184 (6)	0.0168 (6)	0.0275 (7)	−0.0032 (5)	0.0055 (5)	−0.0024 (5)
N1	0.0190 (7)	0.0155 (6)	0.0160 (7)	−0.0029 (5)	−0.0002 (6)	0.0004 (5)
C1	0.0180 (8)	0.0181 (8)	0.0140 (7)	0.0007 (6)	−0.0024 (6)	0.0014 (6)
C2	0.0221 (9)	0.0200 (8)	0.0162 (8)	−0.0006 (7)	0.0005 (6)	0.0016 (6)
C3	0.0290 (9)	0.0226 (8)	0.0159 (8)	0.0028 (7)	0.0027 (7)	−0.0016 (6)
C4	0.0368 (11)	0.0164 (7)	0.0182 (8)	−0.0004 (7)	−0.0015 (7)	−0.0030 (6)
C5	0.0274 (10)	0.0206 (8)	0.0207 (9)	−0.0054 (7)	−0.0015 (7)	0.0005 (6)
C6	0.0207 (8)	0.0197 (8)	0.0184 (8)	−0.0021 (7)	−0.0011 (6)	0.0010 (6)
C7	0.0201 (8)	0.0211 (8)	0.0246 (9)	−0.0043 (7)	0.0004 (7)	0.0036 (7)
C8	0.0256 (9)	0.0273 (9)	0.0202 (9)	−0.0011 (8)	0.0063 (7)	0.0028 (7)
C9	0.0274 (9)	0.0174 (8)	0.0170 (9)	0.0054 (7)	0.0006 (7)	−0.0002 (6)
C10	0.0431 (12)	0.0209 (8)	0.0193 (9)	0.0032 (8)	0.0008 (8)	0.0016 (7)
C11	0.0550 (14)	0.0202 (8)	0.0182 (9)	0.0059 (9)	−0.0107 (9)	−0.0026 (7)
C12	0.0351 (11)	0.0207 (8)	0.0312 (10)	0.0034 (8)	−0.0148 (8)	−0.0066 (7)
C13	0.0241 (9)	0.0188 (8)	0.0274 (9)	0.0026 (7)	−0.0063 (7)	−0.0038 (7)
C14	0.0207 (8)	0.0139 (7)	0.0190 (8)	0.0045 (6)	−0.0030 (6)	−0.0015 (6)
C15	0.0161 (8)	0.0165 (8)	0.0186 (8)	−0.0018 (6)	0.0016 (6)	0.0000 (6)
C16	0.0183 (8)	0.0159 (7)	0.0153 (8)	−0.0010 (6)	0.0009 (6)	0.0009 (6)
C17	0.0265 (9)	0.0170 (8)	0.0150 (8)	−0.0011 (7)	0.0000 (7)	0.0008 (6)
C18	0.0226 (8)	0.0164 (8)	0.0193 (8)	−0.0047 (7)	−0.0037 (6)	0.0015 (6)
C19	0.0325 (11)	0.0230 (9)	0.0284 (10)	−0.0040 (7)	0.0135 (8)	0.0004 (7)
C20	0.0284 (9)	0.0196 (8)	0.0284 (10)	0.0000 (7)	−0.0063 (8)	0.0029 (7)
O2	0.0246 (7)	0.0201 (6)	0.0247 (7)	0.0006 (5)	−0.0032 (5)	−0.0066 (5)
O3	0.0331 (8)	0.0486 (9)	0.0481 (10)	−0.0244 (7)	0.0155 (7)	−0.0238 (8)
O4	0.0283 (7)	0.0177 (6)	0.0275 (7)	−0.0019 (5)	0.0024 (5)	−0.0047 (5)
C21	0.0213 (8)	0.0209 (8)	0.0166 (8)	−0.0017 (7)	−0.0031 (6)	0.0000 (6)
C22	0.0189 (8)	0.0170 (7)	0.0143 (7)	0.0011 (6)	−0.0026 (6)	0.0025 (6)
C23	0.0188 (8)	0.0187 (8)	0.0164 (8)	0.0022 (6)	0.0006 (6)	−0.0012 (6)
C24	0.0168 (8)	0.0186 (8)	0.0201 (8)	−0.0013 (7)	−0.0005 (6)	0.0010 (6)
C25	0.0221 (8)	0.0154 (7)	0.0172 (8)	0.0015 (7)	−0.0023 (6)	0.0010 (6)
C26	0.0220 (9)	0.0211 (8)	0.0183 (8)	0.0051 (7)	0.0027 (7)	−0.0011 (6)
C27	0.0170 (8)	0.0228 (8)	0.0167 (8)	0.0012 (7)	0.0007 (6)	0.0034 (6)
C28	0.0256 (9)	0.0198 (8)	0.0347 (10)	−0.0028 (8)	−0.0049 (8)	−0.0046 (7)
O1W	0.0214 (7)	0.0192 (6)	0.0687 (11)	−0.0040 (6)	−0.0012 (7)	−0.0097 (6)

### (3-{2-Hydroxytricyclo[9.4.0.03,8]pentadeca-1(11),3,5,7,12,14-hexaen-2-yl}propyl)dimethylazanium 4-methoxybenzoate monohydrate (I) Geometric parameters (Å, º)

**Table:**

O1—C15	1.427 (2)	C15—C16	1.553 (2)
O1—H1O	0.85 (3)	C16—C17	1.532 (2)
N1—C19	1.482 (2)	C16—H16B	0.9900
N1—C20	1.492 (2)	C16—H16A	0.9900
N1—C18	1.507 (2)	C17—C18	1.517 (2)
N1—H1N	0.94 (2)	C17—H17A	0.9900
C1—C2	1.389 (2)	C17—H17B	0.9900
C1—C6	1.407 (2)	C18—H18A	0.9900
C1—C15	1.540 (2)	C18—H18B	0.9900
C2—C3	1.387 (2)	C19—H19A	0.9800
C2—H2	0.9500	C19—H19B	0.9800
C3—C4	1.389 (3)	C19—H19C	0.9800
C3—H3	0.9500	C20—H20A	0.9800
C4—C5	1.380 (3)	C20—H20B	0.9800
C4—H4	0.9500	C20—H20C	0.9800
C5—C6	1.397 (2)	O2—C21	1.267 (2)
C5—H5	0.9500	O3—C21	1.243 (2)
C6—C7	1.500 (3)	O4—C25	1.364 (2)
C7—C8	1.524 (3)	O4—C28	1.428 (2)
C7—H7A	0.9900	C21—C22	1.504 (2)
C7—H7B	0.9900	C22—C23	1.392 (2)
C8—C9	1.521 (3)	C22—C27	1.401 (2)
C8—H8A	0.9900	C23—C24	1.392 (2)
C8—H8B	0.9900	C23—H23	0.9500
C9—C14	1.401 (3)	C24—C25	1.393 (2)
C9—C10	1.403 (3)	C24—H24	0.9500
C10—C11	1.381 (3)	C25—C26	1.396 (3)
C10—H10	0.9500	C26—C27	1.375 (2)
C11—C12	1.374 (3)	C26—H26	0.9500
C11—H11	0.9500	C27—H27	0.9500
C12—C13	1.391 (3)	C28—H28A	0.9800
C12—H12	0.9500	C28—H28B	0.9800
C13—C14	1.407 (2)	C28—H28C	0.9800
C13—H13	0.9500	O1W—H1W1	0.84 (3)
C14—C15	1.545 (2)	O1W—H2W1	0.90 (3)
			
C15—O1—H1O	109.4 (18)	C14—C15—C16	110.67 (13)
C19—N1—C20	110.48 (14)	C17—C16—C15	111.99 (14)
C19—N1—C18	112.39 (14)	C17—C16—H16B	109.2
C20—N1—C18	109.28 (13)	C15—C16—H16B	109.2
C19—N1—H1N	104.0 (13)	C17—C16—H16A	109.2
C20—N1—H1N	108.3 (13)	C15—C16—H16A	109.2
C18—N1—H1N	112.2 (13)	H16B—C16—H16A	107.9
C2—C1—C6	118.54 (15)	C18—C17—C16	115.12 (14)
C2—C1—C15	119.39 (15)	C18—C17—H17A	108.5
C6—C1—C15	122.07 (15)	C16—C17—H17A	108.5
C3—C2—C1	121.84 (16)	C18—C17—H17B	108.5
C3—C2—H2	119.1	C16—C17—H17B	108.5
C1—C2—H2	119.1	H17A—C17—H17B	107.5
C2—C3—C4	119.81 (17)	N1—C18—C17	115.72 (14)
C2—C3—H3	120.1	N1—C18—H18A	108.4
C4—C3—H3	120.1	C17—C18—H18A	108.4
C5—C4—C3	118.82 (16)	N1—C18—H18B	108.4
C5—C4—H4	120.6	C17—C18—H18B	108.4
C3—C4—H4	120.6	H18A—C18—H18B	107.4
C4—C5—C6	122.16 (17)	N1—C19—H19A	109.5
C4—C5—H5	118.9	N1—C19—H19B	109.5
C6—C5—H5	118.9	H19A—C19—H19B	109.5
C5—C6—C1	118.81 (16)	N1—C19—H19C	109.5
C5—C6—C7	119.64 (15)	H19A—C19—H19C	109.5
C1—C6—C7	121.55 (15)	H19B—C19—H19C	109.5
C6—C7—C8	113.10 (15)	N1—C20—H20A	109.5
C6—C7—H7A	109.0	N1—C20—H20B	109.5
C8—C7—H7A	109.0	H20A—C20—H20B	109.5
C6—C7—H7B	109.0	N1—C20—H20C	109.5
C8—C7—H7B	109.0	H20A—C20—H20C	109.5
H7A—C7—H7B	107.8	H20B—C20—H20C	109.5
C9—C8—C7	121.58 (15)	C25—O4—C28	117.67 (14)
C9—C8—H8A	106.9	O3—C21—O2	124.79 (16)
C7—C8—H8A	106.9	O3—C21—C22	117.31 (16)
C9—C8—H8B	106.9	O2—C21—C22	117.89 (15)
C7—C8—H8B	106.9	C23—C22—C27	118.31 (15)
H8A—C8—H8B	106.7	C23—C22—C21	122.41 (15)
C14—C9—C10	118.31 (17)	C27—C22—C21	119.23 (15)
C14—C9—C8	128.30 (16)	C24—C23—C22	121.28 (16)
C10—C9—C8	113.26 (16)	C24—C23—H23	119.4
C11—C10—C9	122.71 (19)	C22—C23—H23	119.4
C11—C10—H10	118.6	C23—C24—C25	119.45 (15)
C9—C10—H10	118.6	C23—C24—H24	120.3
C12—C11—C10	119.06 (18)	C25—C24—H24	120.3
C12—C11—H11	120.5	O4—C25—C24	125.17 (15)
C10—C11—H11	120.5	O4—C25—C26	115.15 (15)
C11—C12—C13	119.64 (18)	C24—C25—C26	119.69 (15)
C11—C12—H12	120.2	C27—C26—C25	120.30 (16)
C13—C12—H12	120.2	C27—C26—H26	119.9
C12—C13—C14	121.97 (19)	C25—C26—H26	119.9
C12—C13—H13	119.0	C26—C27—C22	120.96 (16)
C14—C13—H13	119.0	C26—C27—H27	119.5
C9—C14—C13	118.22 (16)	C22—C27—H27	119.5
C9—C14—C15	124.09 (15)	O4—C28—H28A	109.5
C13—C14—C15	117.69 (16)	O4—C28—H28B	109.5
O1—C15—C1	105.14 (13)	H28A—C28—H28B	109.5
O1—C15—C14	110.50 (13)	O4—C28—H28C	109.5
C1—C15—C14	109.65 (13)	H28A—C28—H28C	109.5
O1—C15—C16	106.53 (13)	H28B—C28—H28C	109.5
C1—C15—C16	114.15 (13)	H1W1—O1W—H2W1	109 (3)
			
C6—C1—C2—C3	1.7 (2)	C2—C1—C15—C16	−121.82 (16)
C15—C1—C2—C3	−177.45 (15)	C6—C1—C15—C16	59.1 (2)
C1—C2—C3—C4	−1.2 (3)	C9—C14—C15—O1	171.96 (14)
C2—C3—C4—C5	−0.2 (3)	C13—C14—C15—O1	−7.22 (19)
C3—C4—C5—C6	1.0 (3)	C9—C14—C15—C1	56.5 (2)
C4—C5—C6—C1	−0.5 (3)	C13—C14—C15—C1	−122.66 (16)
C4—C5—C6—C7	179.51 (17)	C9—C14—C15—C16	−70.29 (19)
C2—C1—C6—C5	−0.8 (2)	C13—C14—C15—C16	110.54 (16)
C15—C1—C6—C5	178.29 (15)	O1—C15—C16—C17	−73.23 (16)
C2—C1—C6—C7	179.15 (15)	C1—C15—C16—C17	42.34 (19)
C15—C1—C6—C7	−1.8 (2)	C14—C15—C16—C17	166.61 (13)
C5—C6—C7—C8	−112.86 (18)	C15—C16—C17—C18	172.16 (14)
C1—C6—C7—C8	67.2 (2)	C19—N1—C18—C17	−57.8 (2)
C6—C7—C8—C9	−50.9 (2)	C20—N1—C18—C17	179.13 (15)
C7—C8—C9—C14	2.0 (3)	C16—C17—C18—N1	−71.7 (2)
C7—C8—C9—C10	177.79 (17)	O3—C21—C22—C23	−178.82 (18)
C14—C9—C10—C11	3.0 (3)	O2—C21—C22—C23	2.4 (2)
C8—C9—C10—C11	−173.25 (17)	O3—C21—C22—C27	3.6 (2)
C9—C10—C11—C12	−0.6 (3)	O2—C21—C22—C27	−175.24 (15)
C10—C11—C12—C13	−1.2 (3)	C27—C22—C23—C24	0.1 (2)
C11—C12—C13—C14	0.7 (3)	C21—C22—C23—C24	−177.54 (16)
C10—C9—C14—C13	−3.4 (2)	C22—C23—C24—C25	0.6 (2)
C8—C9—C14—C13	172.22 (17)	C28—O4—C25—C24	−7.5 (2)
C10—C9—C14—C15	177.46 (16)	C28—O4—C25—C26	172.93 (15)
C8—C9—C14—C15	−6.9 (3)	C23—C24—C25—O4	179.18 (16)
C12—C13—C14—C9	1.6 (2)	C23—C24—C25—C26	−1.2 (2)
C12—C13—C14—C15	−179.15 (16)	O4—C25—C26—C27	−179.22 (15)
C2—C1—C15—O1	−5.43 (19)	C24—C25—C26—C27	1.2 (3)
C6—C1—C15—O1	175.48 (15)	C25—C26—C27—C22	−0.4 (3)
C2—C1—C15—C14	113.37 (16)	C23—C22—C27—C26	−0.2 (2)
C6—C1—C15—C14	−65.71 (19)	C21—C22—C27—C26	177.53 (15)

### (3-{2-Hydroxytricyclo[9.4.0.03,8]pentadeca-1(11),3,5,7,12,14-hexaen-2-yl}propyl)dimethylazanium 4-methoxybenzoate monohydrate (I) Hydrogen-bond geometry (Å, º)

**Table:**

*D*—H···*A*	*D*—H	H···*A*	*D*···*A*	*D*—H···*A*
O1—H1*O*···O1*W*^i^	0.85 (3)	1.84 (3)	2.6911 (18)	171 (3)
N1—H1*N*···O2	0.94 (2)	1.76 (2)	2.6711 (18)	163 (2)
C18—H18*A*···O1^ii^	0.99	2.50	3.400 (2)	151
C18—H18*A*···O1*W*	0.99	2.66	3.523 (3)	146
C19—H19*C*···O3^iii^	0.98	2.59	3.191 (3)	119
C20—H20*C*···O1*W*	0.98	2.54	3.420 (3)	149
O1*W*—H1*W*1···O3	0.84 (3)	1.81 (3)	2.646 (2)	173 (3)
O1*W*—H2*W*1···O2^ii^	0.90 (3)	1.84 (3)	2.718 (2)	165 (3)

Symmetry codes: (i) *x*−1, *y*, *z*; (ii) *x*+1, *y*, *z*; (iii) *x*−1/2, −*y*+1, *z*+1/2.

### \ (3-{2-Hydroxytricyclo[9.4.0.03,8]pentadeca-1(11),3,5,7,12,14-hexaen-\ 2-yl}propyl)dimethylazanium 3,4-dimethoxybenzoate trihydrate (II) Crystal data

**Table:**

C_20_H_26_NO^+^·C_9_H_9_O_4_^−^·3H_2_O	*F*(000) = 2288
*M**_r_* = 531.63	*D*_x_ = 1.252 Mg m^−^^3^
Monoclinic, *C**c*	Cu *K*α radiation, λ = 1.54178 Å
*a* = 8.6750 (3) Å	Cell parameters from 9909 reflections
*b* = 55.2061 (19) Å	θ = 3.2–74.4°
*c* = 12.3988 (4) Å	µ = 0.74 mm^−^^1^
β = 108.238 (2)°	*T* = 90 K
*V* = 5639.7 (3) Å^3^	Cut block, colourless
*Z* = 8	0.30 × 0.24 × 0.18 mm

### \ (3-{2-Hydroxytricyclo[9.4.0.03,8]pentadeca-1(11),3,5,7,12,14-hexaen-\ 2-yl}propyl)dimethylazanium 3,4-dimethoxybenzoate trihydrate (II) Data collection

**Table:**

Bruker D8 Venture dual source diffractometer	9069 independent reflections
Radiation source: microsource	8333 reflections with *I* > 2σ(*I*)
Detector resolution: 7.41 pixels mm^-1^	*R*_int_ = 0.034
φ and ω scans	θ_max_ = 74.5°, θ_min_ = 1.6°
Absorption correction: multi-scan (*SADABS*; Krause *et al.*, 2015)	*h* = −10→10
*T*_min_ = 0.858, *T*_max_ = 0.982	*k* = −67→68
22593 measured reflections	*l* = −14→15

### \ (3-{2-Hydroxytricyclo[9.4.0.03,8]pentadeca-1(11),3,5,7,12,14-hexaen-\ 2-yl}propyl)dimethylazanium 3,4-dimethoxybenzoate trihydrate (II) Refinement

**Table:**

Refinement on *F*^2^	Hydrogen site location: mixed
Least-squares matrix: full	H atoms treated by a mixture of independent and constrained refinement
*R*[*F*^2^ > 2σ(*F*^2^)] = 0.031	*w* = 1/[σ^2^(*F*_o_^2^) + (0.0311*P*)^2^ + 1.2839*P*] where *P* = (*F*_o_^2^ + 2*F*_c_^2^)/3
*wR*(*F*^2^) = 0.074	(Δ/σ)_max_ = 0.002
*S* = 1.02	Δρ_max_ = 0.17 e Å^−^^3^
9069 reflections	Δρ_min_ = −0.20 e Å^−^^3^
773 parameters	Extinction correction: *SHELXL2019/2* (Sheldrick, 2015b), Fc^*^=kFc[1+0.001xFc^2^λ^3^/sin(2θ)]^-1/4^
160 restraints	Extinction coefficient: 0.00036 (6)
Primary atom site location: structure-invariant direct methods	Absolute structure: Flack *x* obtained from 2935 quotients [(*I*^+^)-(*I*^-^)]/[(*I*^+^)+(*I*^-^)] (Parsons *et al.*, 2013)
Secondary atom site location: difference Fourier map	Absolute structure parameter: 0.00 (7)

### \ (3-{2-Hydroxytricyclo[9.4.0.03,8]pentadeca-1(11),3,5,7,12,14-hexaen-\ 2-yl}propyl)dimethylazanium 3,4-dimethoxybenzoate trihydrate (II) Special details

**Table:**

Experimental. The crystal was mounted using polyisobutene oil on the tip of a fine glass fibre, which was fastened in a copper mounting pin with electrical solder. It was placed directly into the cold gas stream of a liquid-nitrogen based cryostat (Hope, 1994; Parkin & Hope, 1998). Diffraction data were collected with the crystal at 90K, which is standard practice in this laboratory for the majority of flash-cooled crystals.
Geometry. All esds (except the esd in the dihedral angle between two l.s. planes) are estimated using the full covariance matrix. The cell esds are taken into account individually in the estimation of esds in distances, angles and torsion angles; correlations between esds in cell parameters are only used when they are defined by crystal symmetry. An approximate (isotropic) treatment of cell esds is used for estimating esds involving l.s. planes.
Refinement. Refinement progress was checked using *Platon* (Spek, 2020) and by an *R*-tensor (Parkin, 2000). The final model was further checked with the IUCr utility *checkCIF*.

### \ (3-{2-Hydroxytricyclo[9.4.0.03,8]pentadeca-1(11),3,5,7,12,14-hexaen-\ 2-yl}propyl)dimethylazanium 3,4-dimethoxybenzoate trihydrate (II) Fractional atomic coordinates and isotropic or equivalent isotropic displacement parameters (Å^2^)

**Table:**

	*x*	*y*	*z*	*U*_iso_*/*U*_eq_	Occ. (<1)
O1A	0.8097 (2)	0.47166 (3)	0.10132 (14)	0.0261 (4)	
H1OA	0.728 (5)	0.4647 (6)	0.104 (3)	0.050 (11)*	
N1A	0.9739 (2)	0.42601 (4)	0.47205 (16)	0.0223 (4)	
H1NA	0.944 (4)	0.4156 (5)	0.404 (3)	0.034 (8)*	
C1A	0.7195 (3)	0.51236 (4)	0.1267 (2)	0.0254 (5)	
C2A	0.6083 (3)	0.50919 (5)	0.0187 (2)	0.0338 (6)	
H2A	0.601812	0.493898	−0.017580	0.041*	
C3A	0.5074 (4)	0.52770 (6)	−0.0370 (3)	0.0478 (8)	
H3A	0.431704	0.525070	−0.110150	0.057*	
C4A	0.5177 (4)	0.55017 (6)	0.0150 (3)	0.0520 (9)	
H4A	0.452519	0.563268	−0.023627	0.062*	
C5A	0.6226 (4)	0.55328 (5)	0.1225 (3)	0.0453 (8)	
H5A	0.625477	0.568544	0.158415	0.054*	
C6A	0.7258 (4)	0.53489 (5)	0.1815 (3)	0.0343 (6)	
C7A	0.8349 (7)	0.5406 (2)	0.3010 (6)	0.0430 (7)	0.723 (8)
H7AA	0.774332	0.535915	0.353525	0.052*	0.723 (8)
H7AB	0.847683	0.558391	0.306159	0.052*	0.723 (8)
C8A	1.0040 (6)	0.52974 (10)	0.3483 (4)	0.0412 (10)	0.723 (8)
H8AA	1.076644	0.542410	0.393608	0.049*	0.723 (8)
H8AB	0.998948	0.516568	0.401305	0.049*	0.723 (8)
C9A	1.0821 (3)	0.51966 (5)	0.2649 (2)	0.0328 (6)	
C7A'	0.8294 (15)	0.5414 (5)	0.3009 (13)	0.0430 (7)	0.277 (8)
H7AC	0.801921	0.558060	0.317951	0.052*	0.277 (8)
H7AD	0.801849	0.530334	0.354952	0.052*	0.277 (8)
C8A'	1.0098 (13)	0.5400 (2)	0.3200 (11)	0.0412 (10)	0.277 (8)
H8AC	1.063586	0.539017	0.403067	0.049*	0.277 (8)
H8AD	1.042800	0.555628	0.294696	0.049*	0.277 (8)
C10A	1.2396 (4)	0.52680 (5)	0.2704 (3)	0.0436 (8)	
H10A	1.289198	0.539784	0.319256	0.052*	
C11A	1.3237 (4)	0.51577 (6)	0.2081 (4)	0.0577 (10)	
H11A	1.430100	0.521015	0.213257	0.069*	
C12A	1.2531 (4)	0.49711 (7)	0.1383 (4)	0.0615 (10)	
H12A	1.309878	0.489321	0.093825	0.074*	
C13A	1.0992 (4)	0.48948 (5)	0.1322 (3)	0.0406 (7)	
H13A	1.052750	0.476225	0.084158	0.049*	
C14A	1.0105 (3)	0.50042 (4)	0.1933 (2)	0.0245 (5)	
C15A	0.8373 (3)	0.49146 (4)	0.17906 (19)	0.0223 (5)	
C16A	0.8133 (3)	0.48197 (4)	0.2894 (2)	0.0237 (5)	
H16A	0.828234	0.495479	0.344260	0.028*	
H16B	0.700971	0.475870	0.272840	0.028*	
C17A	0.9323 (3)	0.46170 (4)	0.3429 (2)	0.0265 (5)	
H17A	0.928203	0.449175	0.284873	0.032*	
H17B	1.043917	0.468334	0.369343	0.032*	
C18A	0.8928 (3)	0.45018 (4)	0.44236 (19)	0.0238 (5)	
H18A	0.929122	0.461037	0.509095	0.029*	
H18B	0.773833	0.448128	0.422774	0.029*	
C19A	0.9150 (3)	0.41316 (5)	0.5565 (2)	0.0318 (6)	
H19A	0.796673	0.411542	0.527029	0.048*	
H19B	0.944868	0.422393	0.627505	0.048*	
H19C	0.964507	0.397033	0.570658	0.048*	
C20A	1.1528 (3)	0.42766 (5)	0.5104 (2)	0.0364 (6)	
H20A	1.189226	0.433926	0.448517	0.055*	
H20B	1.199354	0.411546	0.532492	0.055*	
H20C	1.188559	0.438616	0.575741	0.055*	
O2A	0.9079 (2)	0.39435 (3)	0.29857 (14)	0.0288 (4)	
O3A	0.6631 (2)	0.40818 (3)	0.28617 (14)	0.0253 (4)	
O4A	0.8017 (2)	0.32516 (3)	0.01210 (14)	0.0276 (4)	
O5A	0.4946 (2)	0.32461 (3)	−0.08854 (13)	0.0251 (4)	
C21A	0.7548 (3)	0.39383 (4)	0.25680 (19)	0.0220 (5)	
C22A	0.6805 (3)	0.37502 (4)	0.16730 (19)	0.0201 (5)	
C23A	0.7816 (3)	0.35872 (4)	0.13490 (19)	0.0216 (5)	
H23A	0.895721	0.359291	0.170702	0.026*	
C24A	0.7158 (3)	0.34177 (4)	0.05087 (19)	0.0221 (5)	
C25A	0.5467 (3)	0.34135 (4)	−0.00328 (19)	0.0214 (5)	
C26A	0.4471 (3)	0.35703 (4)	0.0312 (2)	0.0234 (5)	
H26A	0.332757	0.356322	−0.003274	0.028*	
C27A	0.5145 (3)	0.37388 (4)	0.1167 (2)	0.0230 (5)	
H27A	0.445821	0.384642	0.140285	0.028*	
C28A	0.9746 (3)	0.32550 (5)	0.0603 (2)	0.0296 (6)	
H28A	1.021890	0.313054	0.023889	0.044*	
H28B	1.004536	0.322238	0.141968	0.044*	
H28C	1.015872	0.341455	0.048111	0.044*	
C29A	0.3318 (3)	0.32681 (5)	−0.1622 (2)	0.0328 (6)	
H29A	0.311686	0.343562	−0.189141	0.049*	
H29B	0.256278	0.322455	−0.120829	0.049*	
H29C	0.315510	0.315935	−0.227306	0.049*	
O2B	0.0055 (2)	0.35198 (3)	0.65098 (13)	0.0255 (4)	
O3B	−0.2199 (2)	0.35121 (3)	0.69922 (14)	0.0276 (4)	
O4B	0.2120 (2)	0.42401 (3)	1.06985 (13)	0.0252 (4)	
O5B	0.35057 (19)	0.41657 (3)	0.91879 (13)	0.0240 (4)	
C21B	−0.0785 (3)	0.35866 (4)	0.71262 (19)	0.0221 (5)	
C22B	−0.0065 (3)	0.37649 (4)	0.80631 (18)	0.0203 (5)	
C23B	0.1409 (3)	0.38828 (4)	0.81467 (19)	0.0204 (5)	
H23B	0.193726	0.385270	0.759468	0.024*	
C24B	0.2089 (3)	0.40417 (4)	0.90259 (19)	0.0192 (5)	
C25B	0.1319 (3)	0.40846 (4)	0.98534 (19)	0.0218 (5)	
C26B	−0.0135 (3)	0.39715 (4)	0.9766 (2)	0.0241 (5)	
H26B	−0.066450	0.400164	1.031777	0.029*	
C27B	−0.0826 (3)	0.38130 (4)	0.8868 (2)	0.0240 (5)	
H27B	−0.183243	0.373717	0.880850	0.029*	
C28B	0.4461 (3)	0.41039 (5)	0.8472 (2)	0.0284 (5)	
H28D	0.386712	0.414694	0.768475	0.043*	
H28E	0.468079	0.392938	0.852269	0.043*	
H28F	0.548977	0.419289	0.871932	0.043*	
C29B	0.1497 (4)	0.42658 (5)	1.1636 (2)	0.0327 (6)	
H29D	0.040051	0.433412	1.136581	0.049*	
H29E	0.220504	0.437408	1.220407	0.049*	
H29F	0.146086	0.410670	1.197751	0.049*	
O1B	−0.0088 (2)	0.27936 (3)	0.84148 (14)	0.0249 (4)	
H1OB	−0.099 (5)	0.2875 (6)	0.828 (3)	0.049 (10)*	
N1B	−0.1886 (2)	0.32466 (4)	0.47886 (16)	0.0226 (4)	
H1NB	−0.124 (3)	0.3328 (5)	0.550 (2)	0.021 (6)*	
C1B	−0.0970 (3)	0.23739 (4)	0.8230 (2)	0.0226 (5)	
C2B	−0.1097 (3)	0.24038 (5)	0.9320 (2)	0.0280 (5)	
H2B	−0.087797	0.255825	0.967240	0.034*	
C3B	−0.1532 (3)	0.22148 (5)	0.9899 (2)	0.0353 (6)	
H3B	−0.161545	0.224032	1.063669	0.042*	
C4B	−0.1845 (3)	0.19891 (5)	0.9399 (3)	0.0364 (7)	
H4B	−0.212209	0.185725	0.979475	0.044*	
C5B	−0.1751 (3)	0.19572 (5)	0.8317 (2)	0.0343 (6)	
H5B	−0.198988	0.180227	0.797139	0.041*	
C6B	−0.1315 (3)	0.21454 (4)	0.7710 (2)	0.0271 (5)	
C7B	−0.1254 (7)	0.20877 (14)	0.6523 (4)	0.0334 (6)	0.794 (10)
H7BA	−0.234262	0.212447	0.598930	0.040*	0.794 (10)
H7BB	−0.110401	0.191034	0.649269	0.040*	0.794 (10)
C8B	−0.0001 (4)	0.22072 (10)	0.6031 (3)	0.0308 (9)	0.794 (10)
H8BA	0.039106	0.208199	0.560842	0.037*	0.794 (10)
H8BB	−0.057133	0.233197	0.547578	0.037*	0.794 (10)
C9B	0.1457 (3)	0.23241 (5)	0.6868 (2)	0.0273 (5)	
C7B'	−0.136 (2)	0.2077 (5)	0.6516 (14)	0.0334 (6)	0.206 (10)
H7BC	−0.218932	0.217595	0.596256	0.040*	0.206 (10)
H7BD	−0.167884	0.190459	0.637791	0.040*	0.206 (10)
C8B'	0.0286 (17)	0.2115 (3)	0.6328 (14)	0.0308 (9)	0.206 (10)
H8BC	0.006565	0.212792	0.549693	0.037*	0.206 (10)
H8BD	0.090553	0.196284	0.656675	0.037*	0.206 (10)
C10B	0.2992 (3)	0.22630 (5)	0.6836 (2)	0.0302 (6)	
H10B	0.310033	0.213517	0.635197	0.036*	
C11B	0.4373 (3)	0.23822 (5)	0.7488 (3)	0.0372 (7)	
H11B	0.541280	0.233658	0.745478	0.045*	
C12B	0.4212 (3)	0.25691 (5)	0.8188 (3)	0.0401 (7)	
H12B	0.514461	0.265297	0.864353	0.048*	
C13B	0.2683 (3)	0.26334 (5)	0.8221 (2)	0.0302 (6)	
H13B	0.258570	0.276233	0.870363	0.036*	
C14B	0.1286 (3)	0.25148 (4)	0.75701 (19)	0.0217 (5)	
C15B	−0.0369 (3)	0.25886 (4)	0.76773 (19)	0.0213 (5)	
C16B	−0.1633 (3)	0.26664 (4)	0.65459 (19)	0.0218 (5)	
H16C	−0.190054	0.252546	0.602651	0.026*	
H16D	−0.264023	0.271806	0.669456	0.026*	
C17B	−0.1019 (3)	0.28741 (4)	0.5966 (2)	0.0235 (5)	
H17C	−0.034510	0.298558	0.655019	0.028*	
H17D	−0.033210	0.280764	0.553196	0.028*	
C18B	−0.2434 (3)	0.30135 (4)	0.51667 (19)	0.0218 (5)	
H18C	−0.296440	0.291225	0.449361	0.026*	
H18D	−0.324476	0.304823	0.555782	0.026*	
C19B	−0.3287 (3)	0.34098 (5)	0.4246 (2)	0.0295 (5)	
H19D	−0.288277	0.356619	0.407438	0.044*	
H19E	−0.392125	0.343457	0.476692	0.044*	
H19F	−0.397610	0.333581	0.354196	0.044*	
C20B	−0.0901 (3)	0.32096 (5)	0.4014 (2)	0.0323 (6)	
H20D	−0.157237	0.313348	0.330950	0.048*	
H20E	0.002483	0.310452	0.438230	0.048*	
H20F	−0.050527	0.336639	0.383928	0.048*	
O1W	0.4417 (3)	0.39328 (4)	0.39210 (18)	0.0413 (5)	
H1W1	0.504 (4)	0.3966 (6)	0.357 (2)	0.052 (9)*	
H2W1	0.489 (4)	0.3906 (7)	0.4585 (16)	0.074 (11)*	
O2W	0.1734 (2)	0.36400 (3)	0.33118 (17)	0.0338 (4)	
H1W2	0.096 (3)	0.3738 (5)	0.315 (3)	0.058 (10)*	
H2W2	0.255 (3)	0.3723 (6)	0.338 (4)	0.081 (11)*	
O3W	0.5334 (3)	0.38545 (4)	0.62643 (19)	0.0413 (5)	
H1W3	0.609 (4)	0.3755 (7)	0.649 (4)	0.091 (13)*	
H2W3	0.451 (3)	0.3769 (7)	0.608 (4)	0.103 (14)*	
O4W	0.2519 (3)	0.36026 (7)	0.5651 (2)	0.0731 (9)	
H1W4	0.185 (4)	0.3580 (9)	0.598 (3)	0.086 (13)*	
H2W4	0.209 (5)	0.3598 (9)	0.4968 (15)	0.103 (14)*	
O5W	0.5522 (2)	0.44426 (4)	0.11870 (17)	0.0382 (5)	
H1W5	0.467 (3)	0.4390 (6)	0.072 (2)	0.059 (10)*	
H2W5	0.576 (4)	0.4335 (5)	0.168 (2)	0.066 (10)*	
O6W	−0.2896 (2)	0.30734 (3)	0.78184 (16)	0.0319 (4)	
H1W6	−0.320 (4)	0.3101 (6)	0.838 (2)	0.053 (9)*	
H2W6	−0.258 (4)	0.3206 (4)	0.765 (3)	0.048 (9)*	

### \ (3-{2-Hydroxytricyclo[9.4.0.03,8]pentadeca-1(11),3,5,7,12,14-hexaen-\ 2-yl}propyl)dimethylazanium 3,4-dimethoxybenzoate trihydrate (II) Atomic displacement parameters (Å^2^)

**Table:**

	*U*^11^	*U*^22^	*U*^33^	*U*^12^	*U*^13^	*U*^23^
O1A	0.0294 (10)	0.0231 (9)	0.0283 (9)	−0.0036 (7)	0.0129 (8)	−0.0060 (7)
N1A	0.0234 (10)	0.0216 (10)	0.0208 (9)	0.0012 (8)	0.0053 (8)	−0.0004 (8)
C1A	0.0234 (12)	0.0263 (13)	0.0328 (13)	0.0007 (9)	0.0178 (11)	0.0054 (10)
C2A	0.0224 (13)	0.0410 (16)	0.0399 (15)	−0.0023 (11)	0.0124 (12)	0.0121 (12)
C3A	0.0215 (14)	0.062 (2)	0.061 (2)	0.0021 (13)	0.0142 (14)	0.0290 (17)
C4A	0.0303 (16)	0.048 (2)	0.089 (3)	0.0166 (13)	0.0340 (18)	0.0377 (18)
C5A	0.0459 (18)	0.0313 (16)	0.076 (2)	0.0125 (13)	0.0449 (18)	0.0159 (15)
C6A	0.0405 (15)	0.0232 (13)	0.0515 (16)	0.0027 (11)	0.0324 (13)	0.0074 (11)
C7A	0.073 (2)	0.0212 (15)	0.0458 (16)	−0.0028 (13)	0.0346 (16)	−0.0045 (12)
C8A	0.062 (2)	0.026 (2)	0.039 (2)	−0.0220 (19)	0.0205 (18)	−0.0088 (17)
C9A	0.0352 (14)	0.0308 (14)	0.0304 (13)	−0.0069 (11)	0.0073 (12)	0.0043 (11)
C7A'	0.073 (2)	0.0212 (15)	0.0458 (16)	−0.0028 (13)	0.0346 (16)	−0.0045 (12)
C8A'	0.062 (2)	0.026 (2)	0.039 (2)	−0.0220 (19)	0.0205 (18)	−0.0088 (17)
C10A	0.0396 (17)	0.0304 (16)	0.0526 (18)	−0.0114 (12)	0.0026 (14)	0.0101 (13)
C11A	0.0316 (17)	0.045 (2)	0.101 (3)	−0.0067 (14)	0.0282 (19)	0.0127 (19)
C12A	0.045 (2)	0.048 (2)	0.110 (3)	−0.0027 (16)	0.052 (2)	−0.007 (2)
C13A	0.0372 (16)	0.0290 (15)	0.066 (2)	−0.0019 (12)	0.0315 (15)	−0.0049 (14)
C14A	0.0243 (12)	0.0210 (12)	0.0308 (12)	−0.0014 (9)	0.0124 (10)	0.0072 (9)
C15A	0.0249 (12)	0.0202 (12)	0.0248 (11)	−0.0008 (9)	0.0119 (10)	−0.0027 (9)
C16A	0.0274 (13)	0.0192 (12)	0.0279 (12)	−0.0025 (9)	0.0135 (10)	−0.0020 (9)
C17A	0.0238 (12)	0.0269 (13)	0.0306 (13)	−0.0003 (10)	0.0112 (11)	0.004 (1)
C18A	0.0261 (13)	0.0224 (12)	0.0242 (12)	0.0019 (9)	0.0096 (10)	−0.0023 (9)
C19A	0.0391 (15)	0.0285 (14)	0.0306 (13)	0.0075 (11)	0.0150 (12)	0.0077 (10)
C20A	0.0238 (13)	0.0389 (16)	0.0398 (15)	0.0006 (11)	0.0006 (12)	−0.0020 (12)
O2A	0.0218 (9)	0.0309 (10)	0.0308 (9)	−0.0011 (7)	0.0040 (7)	−0.0070 (7)
O3A	0.0235 (8)	0.0270 (9)	0.0270 (8)	0.0010 (7)	0.0100 (7)	−0.0021 (7)
O4A	0.0221 (9)	0.0293 (10)	0.0282 (9)	0.0045 (7)	0.0033 (7)	−0.0058 (7)
O5A	0.0232 (9)	0.0253 (9)	0.0224 (8)	0.0020 (7)	0.0007 (7)	−0.0019 (7)
C21A	0.0229 (12)	0.0225 (12)	0.0210 (11)	−0.0020 (9)	0.0073 (9)	0.0029 (9)
C22A	0.0205 (11)	0.0214 (11)	0.0182 (10)	−0.0010 (9)	0.0060 (9)	0.0020 (9)
C23A	0.0184 (11)	0.0226 (12)	0.0221 (11)	−0.0001 (9)	0.0040 (9)	0.0038 (9)
C24A	0.0236 (12)	0.0216 (12)	0.0208 (11)	0.0025 (9)	0.0065 (9)	0.0037 (9)
C25A	0.0238 (11)	0.0196 (11)	0.0192 (11)	−0.0002 (9)	0.0043 (9)	0.0020 (9)
C26A	0.0201 (11)	0.0231 (12)	0.0247 (11)	0.0008 (9)	0.0038 (9)	0.0032 (9)
C27A	0.0233 (12)	0.0226 (12)	0.0240 (11)	0.0015 (9)	0.0087 (10)	0.0039 (9)
C28A	0.0213 (12)	0.0321 (14)	0.0321 (13)	0.0053 (10)	0.0035 (11)	−0.0033 (11)
C29A	0.0254 (13)	0.0301 (14)	0.0329 (14)	0.0036 (10)	−0.0054 (11)	−0.0059 (11)
O2B	0.0239 (9)	0.0282 (9)	0.0243 (8)	−0.0038 (7)	0.0071 (7)	−0.0052 (7)
O3B	0.0238 (9)	0.0286 (9)	0.0296 (9)	−0.0066 (7)	0.0071 (7)	−0.0025 (7)
O4B	0.0299 (9)	0.0251 (9)	0.0215 (8)	−0.0023 (7)	0.0094 (7)	−0.0056 (7)
O5B	0.0215 (8)	0.0264 (9)	0.0256 (8)	−0.0055 (7)	0.0097 (7)	−0.0051 (7)
C21B	0.0233 (12)	0.0189 (11)	0.0219 (11)	−0.0004 (9)	0.0041 (9)	0.0049 (9)
C22B	0.0209 (12)	0.0196 (11)	0.0201 (11)	0.0010 (9)	0.0059 (9)	0.0034 (9)
C23B	0.0211 (11)	0.0216 (12)	0.0187 (11)	0.0014 (9)	0.0066 (9)	0.0028 (9)
C24B	0.0188 (11)	0.0182 (11)	0.0201 (11)	0.0015 (8)	0.0055 (9)	0.0037 (8)
C25B	0.0232 (12)	0.0208 (12)	0.0202 (11)	0.0017 (9)	0.0048 (9)	0.0010 (9)
C26B	0.0278 (12)	0.0254 (13)	0.0230 (11)	0.0022 (10)	0.0137 (10)	0.0025 (9)
C27B	0.0211 (12)	0.0248 (12)	0.0275 (12)	−0.0025 (9)	0.0095 (10)	0.0038 (9)
C28B	0.0249 (12)	0.0338 (14)	0.0308 (13)	−0.0058 (10)	0.0147 (11)	−0.0050 (11)
C29B	0.0407 (15)	0.0350 (15)	0.0255 (13)	−0.0041 (12)	0.0150 (12)	−0.0069 (11)
O1B	0.0236 (9)	0.0210 (9)	0.0278 (9)	0.0013 (7)	0.0047 (7)	−0.0052 (7)
N1B	0.0233 (10)	0.0219 (10)	0.0208 (10)	−0.0021 (8)	0.0045 (8)	0.0003 (8)
C1B	0.0144 (10)	0.0210 (12)	0.0288 (12)	0.0024 (8)	0.0014 (9)	0.0036 (9)
C2B	0.0221 (12)	0.0286 (13)	0.0325 (13)	0.0024 (10)	0.0074 (11)	0.0014 (10)
C3B	0.0258 (13)	0.0440 (17)	0.0370 (15)	0.0043 (11)	0.0113 (12)	0.0118 (12)
C4B	0.0205 (13)	0.0354 (16)	0.0524 (17)	0.0003 (10)	0.0102 (12)	0.0161 (13)
C5B	0.0194 (12)	0.0239 (13)	0.0510 (17)	−0.002 (1)	−0.0013 (12)	0.0039 (12)
C6B	0.0183 (11)	0.0237 (12)	0.0320 (12)	0.0013 (9)	−0.0026 (10)	0.0008 (10)
C7B	0.0363 (16)	0.0203 (14)	0.0350 (14)	−0.0037 (11)	−0.0013 (12)	−0.0060 (11)
C8B	0.0253 (16)	0.032 (2)	0.0303 (18)	0.0102 (14)	0.0013 (14)	−0.0078 (15)
C9B	0.0256 (12)	0.0295 (13)	0.0257 (12)	0.0045 (10)	0.0063 (10)	0.0013 (10)
C7B'	0.0363 (16)	0.0203 (14)	0.0350 (14)	−0.0037 (11)	−0.0013 (12)	−0.0060 (11)
C8B'	0.0253 (16)	0.032 (2)	0.0303 (18)	0.0102 (14)	0.0013 (14)	−0.0078 (15)
C10B	0.0289 (13)	0.0293 (14)	0.0342 (13)	0.0075 (10)	0.0123 (11)	0.0045 (11)
C11B	0.0255 (14)	0.0353 (16)	0.0552 (18)	0.0043 (11)	0.0190 (13)	0.0074 (13)
C12B	0.0226 (13)	0.0377 (16)	0.0569 (18)	−0.0090 (11)	0.0078 (13)	−0.0028 (13)
C13B	0.0247 (13)	0.0253 (14)	0.0387 (14)	−0.0015 (10)	0.0071 (11)	−0.0012 (11)
C14B	0.0198 (11)	0.0223 (12)	0.0214 (11)	0.0027 (9)	0.0040 (9)	0.0051 (9)
C15B	0.0218 (11)	0.0195 (11)	0.0203 (11)	0.0002 (9)	0.0034 (9)	−0.0029 (9)
C16B	0.0195 (11)	0.0182 (11)	0.0250 (11)	0.0013 (8)	0.0030 (9)	−0.0001 (9)
C17B	0.0217 (12)	0.0234 (12)	0.0244 (12)	0.0012 (9)	0.0059 (10)	0.0015 (9)
C18B	0.0201 (11)	0.0213 (12)	0.0230 (11)	−0.0021 (9)	0.0054 (9)	0.0000 (9)
C19B	0.0293 (13)	0.0246 (13)	0.0283 (12)	0.001 (1)	0.0000 (11)	0.0034 (10)
C20B	0.0341 (15)	0.0372 (15)	0.0266 (13)	−0.0058 (11)	0.0110 (11)	−0.0007 (11)
O1W	0.0361 (11)	0.0495 (13)	0.0433 (12)	−0.0045 (9)	0.0198 (10)	0.0081 (10)
O2W	0.0261 (10)	0.0304 (10)	0.0406 (11)	0.0033 (8)	0.0044 (9)	−0.0038 (8)
O3W	0.0401 (12)	0.0330 (11)	0.0427 (12)	0.0035 (9)	0.0011 (10)	−0.0047 (9)
O4W	0.0344 (13)	0.142 (3)	0.0407 (14)	−0.0144 (15)	0.0091 (11)	0.0307 (16)
O5W	0.0339 (11)	0.0308 (11)	0.0405 (11)	−0.0124 (8)	−0.0021 (9)	0.0065 (9)
O6W	0.0372 (10)	0.0275 (10)	0.0364 (10)	0.0034 (8)	0.0194 (9)	−0.0023 (8)

### \ (3-{2-Hydroxytricyclo[9.4.0.03,8]pentadeca-1(11),3,5,7,12,14-hexaen-\ 2-yl}propyl)dimethylazanium 3,4-dimethoxybenzoate trihydrate (II) Geometric parameters (Å, º)

**Table:**

O1A—C15A	1.427 (3)	C21B—C22B	1.501 (3)
O1A—H1OA	0.82 (4)	C22B—C27B	1.384 (3)
N1A—C20A	1.477 (3)	C22B—C23B	1.409 (3)
N1A—C19A	1.481 (3)	C23B—C24B	1.380 (3)
N1A—C18A	1.500 (3)	C23B—H23B	0.9500
N1A—H1NA	0.99 (3)	C24B—C25B	1.408 (3)
C1A—C2A	1.395 (4)	C25B—C26B	1.381 (3)
C1A—C6A	1.410 (4)	C26B—C27B	1.396 (3)
C1A—C15A	1.543 (3)	C26B—H26B	0.9500
C2A—C3A	1.381 (4)	C27B—H27B	0.9500
C2A—H2A	0.9500	C28B—H28D	0.9800
C3A—C4A	1.388 (5)	C28B—H28E	0.9800
C3A—H3A	0.9500	C28B—H28F	0.9800
C4A—C5A	1.369 (5)	C29B—H29D	0.9800
C4A—H4A	0.9500	C29B—H29E	0.9800
C5A—C6A	1.399 (4)	C29B—H29F	0.9800
C5A—H5A	0.9500	O1B—C15B	1.427 (3)
C6A—C7A'	1.514 (11)	O1B—H1OB	0.87 (4)
C6A—C7A	1.520 (6)	N1B—C20B	1.486 (3)
C7A—C8A	1.522 (7)	N1B—C19B	1.493 (3)
C7A—H7AA	0.9900	N1B—C18B	1.497 (3)
C7A—H7AB	0.9900	N1B—H1NB	0.99 (3)
C8A—C9A	1.509 (5)	C1B—C2B	1.399 (3)
C8A—H8AA	0.9900	C1B—C6B	1.406 (3)
C8A—H8AB	0.9900	C1B—C15B	1.540 (3)
C9A—C14A	1.399 (4)	C2B—C3B	1.385 (4)
C9A—C10A	1.403 (4)	C2B—H2B	0.9500
C9A—C8A'	1.546 (9)	C3B—C4B	1.380 (4)
C7A'—C8A'	1.509 (12)	C3B—H3B	0.9500
C7A'—H7AC	0.9900	C4B—C5B	1.380 (4)
C7A'—H7AD	0.9900	C4B—H4B	0.9500
C8A'—H8AC	0.9900	C5B—C6B	1.403 (4)
C8A'—H8AD	0.9900	C5B—H5B	0.9500
C10A—C11A	1.361 (5)	C6B—C7B'	1.517 (11)
C10A—H10A	0.9500	C6B—C7B	1.523 (5)
C11A—C12A	1.362 (5)	C7B—C8B	1.551 (5)
C11A—H11A	0.9500	C7B—H7BA	0.9900
C12A—C13A	1.379 (4)	C7B—H7BB	0.9900
C12A—H12A	0.9500	C8B—C9B	1.506 (4)
C13A—C14A	1.376 (4)	C8B—H8BA	0.9900
C13A—H13A	0.9500	C8B—H8BB	0.9900
C14A—C15A	1.539 (3)	C9B—C10B	1.387 (4)
C15A—C16A	1.539 (3)	C9B—C14B	1.404 (3)
C16A—C17A	1.526 (3)	C9B—C8B'	1.545 (10)
C16A—H16A	0.9900	C7B'—C8B'	1.531 (12)
C16A—H16B	0.9900	C7B'—H7BC	0.9900
C17A—C18A	1.520 (3)	C7B'—H7BD	0.9900
C17A—H17A	0.9900	C8B'—H8BC	0.9900
C17A—H17B	0.9900	C8B'—H8BD	0.9900
C18A—H18A	0.9900	C10B—C11B	1.384 (4)
C18A—H18B	0.9900	C10B—H10B	0.9500
C19A—H19A	0.9800	C11B—C12B	1.383 (4)
C19A—H19B	0.9800	C11B—H11B	0.9500
C19A—H19C	0.9800	C12B—C13B	1.386 (4)
C20A—H20A	0.9800	C12B—H12B	0.9500
C20A—H20B	0.9800	C13B—C14B	1.392 (3)
C20A—H20C	0.9800	C13B—H13B	0.9500
O2A—C21A	1.266 (3)	C14B—C15B	1.538 (3)
O3A—C21A	1.254 (3)	C15B—C16B	1.546 (3)
O4A—C24A	1.361 (3)	C16B—C17B	1.535 (3)
O4A—C28A	1.430 (3)	C16B—H16C	0.9900
O5A—C25A	1.370 (3)	C16B—H16D	0.9900
O5A—C29A	1.427 (3)	C17B—C18B	1.523 (3)
C21A—C22A	1.509 (3)	C17B—H17C	0.9900
C22A—C27A	1.381 (3)	C17B—H17D	0.9900
C22A—C23A	1.401 (3)	C18B—H18C	0.9900
C23A—C24A	1.383 (3)	C18B—H18D	0.9900
C23A—H23A	0.9500	C19B—H19D	0.9800
C24A—C25A	1.409 (3)	C19B—H19E	0.9800
C25A—C26A	1.382 (3)	C19B—H19F	0.9800
C26A—C27A	1.394 (3)	C20B—H20D	0.9800
C26A—H26A	0.9500	C20B—H20E	0.9800
C27A—H27A	0.9500	C20B—H20F	0.9800
C28A—H28A	0.9800	O1W—H1W1	0.815 (16)
C28A—H28B	0.9800	O1W—H2W1	0.810 (16)
C28A—H28C	0.9800	O2W—H1W2	0.833 (16)
C29A—H29A	0.9800	O2W—H2W2	0.826 (17)
C29A—H29B	0.9800	O3W—H1W3	0.831 (17)
C29A—H29C	0.9800	O3W—H2W3	0.825 (17)
O2B—C21B	1.265 (3)	O4W—H1W4	0.823 (17)
O3B—C21B	1.255 (3)	O4W—H2W4	0.812 (17)
O4B—C25B	1.364 (3)	O5W—H1W5	0.834 (16)
O4B—C29B	1.433 (3)	O5W—H2W5	0.829 (16)
O5B—C24B	1.366 (3)	O6W—H1W6	0.835 (16)
O5B—C28B	1.432 (3)	O6W—H2W6	0.832 (16)
			
C15A—O1A—H1OA	107 (3)	O3B—C21B—C22B	118.1 (2)
C20A—N1A—C19A	111.2 (2)	O2B—C21B—C22B	118.7 (2)
C20A—N1A—C18A	112.9 (2)	C27B—C22B—C23B	119.0 (2)
C19A—N1A—C18A	111.13 (19)	C27B—C22B—C21B	120.5 (2)
C20A—N1A—H1NA	106.2 (18)	C23B—C22B—C21B	120.5 (2)
C19A—N1A—H1NA	105.8 (17)	C24B—C23B—C22B	120.4 (2)
C18A—N1A—H1NA	109.2 (17)	C24B—C23B—H23B	119.8
C2A—C1A—C6A	118.9 (2)	C22B—C23B—H23B	119.8
C2A—C1A—C15A	118.5 (2)	O5B—C24B—C23B	125.3 (2)
C6A—C1A—C15A	122.4 (2)	O5B—C24B—C25B	114.74 (19)
C3A—C2A—C1A	121.8 (3)	C23B—C24B—C25B	120.0 (2)
C3A—C2A—H2A	119.1	O4B—C25B—C26B	125.3 (2)
C1A—C2A—H2A	119.1	O4B—C25B—C24B	115.0 (2)
C2A—C3A—C4A	119.4 (3)	C26B—C25B—C24B	119.8 (2)
C2A—C3A—H3A	120.3	C25B—C26B—C27B	120.0 (2)
C4A—C3A—H3A	120.3	C25B—C26B—H26B	120.0
C5A—C4A—C3A	119.4 (3)	C27B—C26B—H26B	120.0
C5A—C4A—H4A	120.3	C22B—C27B—C26B	120.9 (2)
C3A—C4A—H4A	120.3	C22B—C27B—H27B	119.6
C4A—C5A—C6A	122.7 (3)	C26B—C27B—H27B	119.6
C4A—C5A—H5A	118.7	O5B—C28B—H28D	109.5
C6A—C5A—H5A	118.7	O5B—C28B—H28E	109.5
C5A—C6A—C1A	117.8 (3)	H28D—C28B—H28E	109.5
C5A—C6A—C7A'	115.2 (9)	O5B—C28B—H28F	109.5
C1A—C6A—C7A'	127.0 (9)	H28D—C28B—H28F	109.5
C5A—C6A—C7A	117.5 (4)	H28E—C28B—H28F	109.5
C1A—C6A—C7A	124.7 (4)	O4B—C29B—H29D	109.5
C6A—C7A—C8A	121.7 (4)	O4B—C29B—H29E	109.5
C6A—C7A—H7AA	106.9	H29D—C29B—H29E	109.5
C8A—C7A—H7AA	106.9	O4B—C29B—H29F	109.5
C6A—C7A—H7AB	106.9	H29D—C29B—H29F	109.5
C8A—C7A—H7AB	106.9	H29E—C29B—H29F	109.5
H7AA—C7A—H7AB	106.7	C15B—O1B—H1OB	109 (2)
C9A—C8A—C7A	117.8 (4)	C20B—N1B—C19B	110.10 (19)
C9A—C8A—H8AA	107.9	C20B—N1B—C18B	112.79 (19)
C7A—C8A—H8AA	107.9	C19B—N1B—C18B	111.48 (19)
C9A—C8A—H8AB	107.9	C20B—N1B—H1NB	110.7 (15)
C7A—C8A—H8AB	107.9	C19B—N1B—H1NB	106.4 (15)
H8AA—C8A—H8AB	107.2	C18B—N1B—H1NB	105.1 (15)
C14A—C9A—C10A	118.5 (3)	C2B—C1B—C6B	118.6 (2)
C14A—C9A—C8A	120.6 (3)	C2B—C1B—C15B	118.6 (2)
C10A—C9A—C8A	120.2 (3)	C6B—C1B—C15B	122.7 (2)
C14A—C9A—C8A'	132.1 (5)	C3B—C2B—C1B	121.9 (3)
C10A—C9A—C8A'	107.3 (5)	C3B—C2B—H2B	119.0
C8A'—C7A'—C6A	114.3 (10)	C1B—C2B—H2B	119.0
C8A'—C7A'—H7AC	108.7	C4B—C3B—C2B	119.6 (3)
C6A—C7A'—H7AC	108.7	C4B—C3B—H3B	120.2
C8A'—C7A'—H7AD	108.7	C2B—C3B—H3B	120.2
C6A—C7A'—H7AD	108.7	C3B—C4B—C5B	119.3 (3)
H7AC—C7A'—H7AD	107.6	C3B—C4B—H4B	120.4
C7A'—C8A'—C9A	120.7 (9)	C5B—C4B—H4B	120.4
C7A'—C8A'—H8AC	107.1	C4B—C5B—C6B	122.3 (3)
C9A—C8A'—H8AC	107.1	C4B—C5B—H5B	118.8
C7A'—C8A'—H8AD	107.1	C6B—C5B—H5B	118.8
C9A—C8A'—H8AD	107.1	C5B—C6B—C1B	118.3 (2)
H8AC—C8A'—H8AD	106.8	C5B—C6B—C7B'	114.2 (9)
C11A—C10A—C9A	122.3 (3)	C1B—C6B—C7B'	127.5 (10)
C11A—C10A—H10A	118.9	C5B—C6B—C7B	117.4 (3)
C9A—C10A—H10A	118.9	C1B—C6B—C7B	124.4 (3)
C10A—C11A—C12A	119.0 (3)	C6B—C7B—C8B	122.1 (3)
C10A—C11A—H11A	120.5	C6B—C7B—H7BA	106.8
C12A—C11A—H11A	120.5	C8B—C7B—H7BA	106.8
C11A—C12A—C13A	120.1 (3)	C6B—C7B—H7BB	106.8
C11A—C12A—H12A	119.9	C8B—C7B—H7BB	106.8
C13A—C12A—H12A	119.9	H7BA—C7B—H7BB	106.6
C14A—C13A—C12A	122.3 (3)	C9B—C8B—C7B	116.9 (3)
C14A—C13A—H13A	118.9	C9B—C8B—H8BA	108.1
C12A—C13A—H13A	118.9	C7B—C8B—H8BA	108.1
C13A—C14A—C9A	117.9 (2)	C9B—C8B—H8BB	108.1
C13A—C14A—C15A	119.3 (2)	C7B—C8B—H8BB	108.1
C9A—C14A—C15A	122.7 (2)	H8BA—C8B—H8BB	107.3
O1A—C15A—C14A	105.94 (19)	C10B—C9B—C14B	119.3 (2)
O1A—C15A—C16A	107.40 (19)	C10B—C9B—C8B	118.9 (3)
C14A—C15A—C16A	114.0 (2)	C14B—C9B—C8B	121.3 (3)
O1A—C15A—C1A	109.87 (19)	C10B—C9B—C8B'	108.2 (6)
C14A—C15A—C1A	107.60 (19)	C14B—C9B—C8B'	130.3 (5)
C16A—C15A—C1A	111.86 (19)	C6B—C7B'—C8B'	112.0 (11)
C17A—C16A—C15A	111.99 (19)	C6B—C7B'—H7BC	109.2
C17A—C16A—H16A	109.2	C8B'—C7B'—H7BC	109.2
C15A—C16A—H16A	109.2	C6B—C7B'—H7BD	109.2
C17A—C16A—H16B	109.2	C8B'—C7B'—H7BD	109.2
C15A—C16A—H16B	109.2	H7BC—C7B'—H7BD	107.9
H16A—C16A—H16B	107.9	C7B'—C8B'—C9B	123.6 (10)
C18A—C17A—C16A	111.4 (2)	C7B'—C8B'—H8BC	106.4
C18A—C17A—H17A	109.3	C9B—C8B'—H8BC	106.4
C16A—C17A—H17A	109.3	C7B'—C8B'—H8BD	106.4
C18A—C17A—H17B	109.3	C9B—C8B'—H8BD	106.4
C16A—C17A—H17B	109.3	H8BC—C8B'—H8BD	106.5
H17A—C17A—H17B	108.0	C11B—C10B—C9B	122.1 (3)
N1A—C18A—C17A	111.35 (19)	C11B—C10B—H10B	118.9
N1A—C18A—H18A	109.4	C9B—C10B—H10B	118.9
C17A—C18A—H18A	109.4	C12B—C11B—C10B	118.8 (3)
N1A—C18A—H18B	109.4	C12B—C11B—H11B	120.6
C17A—C18A—H18B	109.4	C10B—C11B—H11B	120.6
H18A—C18A—H18B	108.0	C11B—C12B—C13B	119.7 (3)
N1A—C19A—H19A	109.5	C11B—C12B—H12B	120.2
N1A—C19A—H19B	109.5	C13B—C12B—H12B	120.2
H19A—C19A—H19B	109.5	C12B—C13B—C14B	122.0 (3)
N1A—C19A—H19C	109.5	C12B—C13B—H13B	119.0
H19A—C19A—H19C	109.5	C14B—C13B—H13B	119.0
H19B—C19A—H19C	109.5	C13B—C14B—C9B	118.1 (2)
N1A—C20A—H20A	109.5	C13B—C14B—C15B	119.5 (2)
N1A—C20A—H20B	109.5	C9B—C14B—C15B	122.4 (2)
H20A—C20A—H20B	109.5	O1B—C15B—C14B	106.77 (19)
N1A—C20A—H20C	109.5	O1B—C15B—C1B	109.81 (19)
H20A—C20A—H20C	109.5	C14B—C15B—C1B	106.86 (18)
H20B—C20A—H20C	109.5	O1B—C15B—C16B	106.96 (18)
C24A—O4A—C28A	117.51 (19)	C14B—C15B—C16B	114.27 (19)
C25A—O5A—C29A	117.25 (19)	C1B—C15B—C16B	112.01 (19)
O3A—C21A—O2A	123.1 (2)	C17B—C16B—C15B	112.40 (19)
O3A—C21A—C22A	119.0 (2)	C17B—C16B—H16C	109.1
O2A—C21A—C22A	117.9 (2)	C15B—C16B—H16C	109.1
C27A—C22A—C23A	119.9 (2)	C17B—C16B—H16D	109.1
C27A—C22A—C21A	120.7 (2)	C15B—C16B—H16D	109.1
C23A—C22A—C21A	119.4 (2)	H16C—C16B—H16D	107.9
C24A—C23A—C22A	120.2 (2)	C18B—C17B—C16B	110.81 (19)
C24A—C23A—H23A	119.9	C18B—C17B—H17C	109.5
C22A—C23A—H23A	119.9	C16B—C17B—H17C	109.5
O4A—C24A—C23A	125.4 (2)	C18B—C17B—H17D	109.5
O4A—C24A—C25A	115.1 (2)	C16B—C17B—H17D	109.5
C23A—C24A—C25A	119.5 (2)	H17C—C17B—H17D	108.1
O5A—C25A—C26A	125.0 (2)	N1B—C18B—C17B	111.57 (19)
O5A—C25A—C24A	115.0 (2)	N1B—C18B—H18C	109.3
C26A—C25A—C24A	120.0 (2)	C17B—C18B—H18C	109.3
C25A—C26A—C27A	119.9 (2)	N1B—C18B—H18D	109.3
C25A—C26A—H26A	120.0	C17B—C18B—H18D	109.3
C27A—C26A—H26A	120.0	H18C—C18B—H18D	108.0
C22A—C27A—C26A	120.4 (2)	N1B—C19B—H19D	109.5
C22A—C27A—H27A	119.8	N1B—C19B—H19E	109.5
C26A—C27A—H27A	119.8	H19D—C19B—H19E	109.5
O4A—C28A—H28A	109.5	N1B—C19B—H19F	109.5
O4A—C28A—H28B	109.5	H19D—C19B—H19F	109.5
H28A—C28A—H28B	109.5	H19E—C19B—H19F	109.5
O4A—C28A—H28C	109.5	N1B—C20B—H20D	109.5
H28A—C28A—H28C	109.5	N1B—C20B—H20E	109.5
H28B—C28A—H28C	109.5	H20D—C20B—H20E	109.5
O5A—C29A—H29A	109.5	N1B—C20B—H20F	109.5
O5A—C29A—H29B	109.5	H20D—C20B—H20F	109.5
H29A—C29A—H29B	109.5	H20E—C20B—H20F	109.5
O5A—C29A—H29C	109.5	H1W1—O1W—H2W1	112 (3)
H29A—C29A—H29C	109.5	H1W2—O2W—H2W2	105 (3)
H29B—C29A—H29C	109.5	H1W3—O3W—H2W3	104 (3)
C25B—O4B—C29B	117.04 (19)	H1W4—O4W—H2W4	111 (3)
C24B—O5B—C28B	117.26 (18)	H1W5—O5W—H2W5	103 (3)
O3B—C21B—O2B	123.3 (2)	H1W6—O6W—H2W6	106 (2)
			
C6A—C1A—C2A—C3A	1.6 (4)	O3B—C21B—C22B—C27B	10.7 (3)
C15A—C1A—C2A—C3A	−175.1 (2)	O2B—C21B—C22B—C27B	−169.5 (2)
C1A—C2A—C3A—C4A	0.8 (4)	O3B—C21B—C22B—C23B	−170.3 (2)
C2A—C3A—C4A—C5A	−2.8 (4)	O2B—C21B—C22B—C23B	9.5 (3)
C3A—C4A—C5A—C6A	2.5 (4)	C27B—C22B—C23B—C24B	0.7 (3)
C4A—C5A—C6A—C1A	−0.1 (4)	C21B—C22B—C23B—C24B	−178.3 (2)
C4A—C5A—C6A—C7A'	−178.1 (10)	C28B—O5B—C24B—C23B	−8.1 (3)
C4A—C5A—C6A—C7A	−179.2 (5)	C28B—O5B—C24B—C25B	171.3 (2)
C2A—C1A—C6A—C5A	−1.9 (4)	C22B—C23B—C24B—O5B	−179.9 (2)
C15A—C1A—C6A—C5A	174.7 (2)	C22B—C23B—C24B—C25B	0.7 (3)
C2A—C1A—C6A—C7A'	175.8 (11)	C29B—O4B—C25B—C26B	7.6 (3)
C15A—C1A—C6A—C7A'	−7.6 (12)	C29B—O4B—C25B—C24B	−172.3 (2)
C2A—C1A—C6A—C7A	177.1 (5)	O5B—C24B—C25B—O4B	−1.0 (3)
C15A—C1A—C6A—C7A	−6.3 (6)	C23B—C24B—C25B—O4B	178.4 (2)
C5A—C6A—C7A—C8A	−146.0 (7)	O5B—C24B—C25B—C26B	179.1 (2)
C1A—C6A—C7A—C8A	35.0 (12)	C23B—C24B—C25B—C26B	−1.5 (3)
C6A—C7A—C8A—C9A	18.7 (12)	O4B—C25B—C26B—C27B	−179.1 (2)
C7A—C8A—C9A—C14A	−61.2 (7)	C24B—C25B—C26B—C27B	0.8 (3)
C7A—C8A—C9A—C10A	128.9 (6)	C23B—C22B—C27B—C26B	−1.4 (3)
C5A—C6A—C7A'—C8A'	−119.5 (15)	C21B—C22B—C27B—C26B	177.6 (2)
C1A—C6A—C7A'—C8A'	63 (2)	C25B—C26B—C27B—C22B	0.7 (4)
C6A—C7A'—C8A'—C9A	−38 (3)	C6B—C1B—C2B—C3B	0.7 (4)
C14A—C9A—C8A'—C7A'	1 (2)	C15B—C1B—C2B—C3B	−176.0 (2)
C10A—C9A—C8A'—C7A'	163.6 (15)	C1B—C2B—C3B—C4B	0.4 (4)
C14A—C9A—C10A—C11A	0.4 (4)	C2B—C3B—C4B—C5B	−1.4 (4)
C8A—C9A—C10A—C11A	170.5 (4)	C3B—C4B—C5B—C6B	1.3 (4)
C8A'—C9A—C10A—C11A	−165.2 (6)	C4B—C5B—C6B—C1B	−0.2 (4)
C9A—C10A—C11A—C12A	−0.2 (5)	C4B—C5B—C6B—C7B'	−177.3 (11)
C10A—C11A—C12A—C13A	−0.6 (6)	C4B—C5B—C6B—C7B	180.0 (4)
C11A—C12A—C13A—C14A	1.2 (6)	C2B—C1B—C6B—C5B	−0.8 (3)
C12A—C13A—C14A—C9A	−1.0 (5)	C15B—C1B—C6B—C5B	175.8 (2)
C12A—C13A—C14A—C15A	178.0 (3)	C2B—C1B—C6B—C7B'	175.8 (12)
C10A—C9A—C14A—C13A	0.2 (4)	C15B—C1B—C6B—C7B'	−7.6 (12)
C8A—C9A—C14A—C13A	−169.9 (3)	C2B—C1B—C6B—C7B	179.0 (4)
C8A'—C9A—C14A—C13A	161.6 (8)	C15B—C1B—C6B—C7B	−4.5 (5)
C10A—C9A—C14A—C15A	−178.8 (2)	C5B—C6B—C7B—C8B	−145.8 (5)
C8A—C9A—C14A—C15A	11.2 (4)	C1B—C6B—C7B—C8B	34.5 (9)
C8A'—C9A—C14A—C15A	−17.4 (9)	C6B—C7B—C8B—C9B	17.7 (9)
C13A—C14A—C15A—O1A	1.9 (3)	C7B—C8B—C9B—C10B	128.6 (5)
C9A—C14A—C15A—O1A	−179.2 (2)	C7B—C8B—C9B—C14B	−59.8 (6)
C13A—C14A—C15A—C16A	119.7 (3)	C5B—C6B—C7B'—C8B'	−122.2 (16)
C9A—C14A—C15A—C16A	−61.3 (3)	C1B—C6B—C7B'—C8B'	61 (2)
C13A—C14A—C15A—C1A	−115.6 (3)	C6B—C7B'—C8B'—C9B	−35 (3)
C9A—C14A—C15A—C1A	63.3 (3)	C10B—C9B—C8B'—C7B'	160.9 (18)
C2A—C1A—C15A—O1A	0.7 (3)	C14B—C9B—C8B'—C7B'	−2 (3)
C6A—C1A—C15A—O1A	−175.9 (2)	C14B—C9B—C10B—C11B	0.8 (4)
C2A—C1A—C15A—C14A	115.6 (2)	C8B—C9B—C10B—C11B	172.6 (3)
C6A—C1A—C15A—C14A	−61.0 (3)	C8B'—C9B—C10B—C11B	−164.0 (8)
C2A—C1A—C15A—C16A	−118.4 (2)	C9B—C10B—C11B—C12B	−0.3 (4)
C6A—C1A—C15A—C16A	64.9 (3)	C10B—C11B—C12B—C13B	−0.2 (4)
O1A—C15A—C16A—C17A	60.7 (3)	C11B—C12B—C13B—C14B	0.2 (5)
C14A—C15A—C16A—C17A	−56.3 (3)	C12B—C13B—C14B—C9B	0.3 (4)
C1A—C15A—C16A—C17A	−178.7 (2)	C12B—C13B—C14B—C15B	177.8 (3)
C15A—C16A—C17A—C18A	−172.24 (19)	C10B—C9B—C14B—C13B	−0.8 (4)
C20A—N1A—C18A—C17A	63.2 (3)	C8B—C9B—C14B—C13B	−172.3 (3)
C19A—N1A—C18A—C17A	−171.1 (2)	C8B'—C9B—C14B—C13B	160.2 (10)
C16A—C17A—C18A—N1A	162.68 (19)	C10B—C9B—C14B—C15B	−178.2 (2)
O3A—C21A—C22A—C27A	1.3 (3)	C8B—C9B—C14B—C15B	10.2 (4)
O2A—C21A—C22A—C27A	−178.6 (2)	C8B'—C9B—C14B—C15B	−17.2 (11)
O3A—C21A—C22A—C23A	−179.2 (2)	C13B—C14B—C15B—O1B	5.2 (3)
O2A—C21A—C22A—C23A	0.9 (3)	C9B—C14B—C15B—O1B	−177.4 (2)
C27A—C22A—C23A—C24A	1.2 (3)	C13B—C14B—C15B—C1B	−112.3 (2)
C21A—C22A—C23A—C24A	−178.3 (2)	C9B—C14B—C15B—C1B	65.1 (3)
C28A—O4A—C24A—C23A	−1.4 (3)	C13B—C14B—C15B—C16B	123.2 (2)
C28A—O4A—C24A—C25A	177.2 (2)	C9B—C14B—C15B—C16B	−59.4 (3)
C22A—C23A—C24A—O4A	179.7 (2)	C2B—C1B—C15B—O1B	−2.1 (3)
C22A—C23A—C24A—C25A	1.2 (3)	C6B—C1B—C15B—O1B	−178.6 (2)
C29A—O5A—C25A—C26A	13.6 (3)	C2B—C1B—C15B—C14B	113.4 (2)
C29A—O5A—C25A—C24A	−166.7 (2)	C6B—C1B—C15B—C14B	−63.2 (3)
O4A—C24A—C25A—O5A	−1.3 (3)	C2B—C1B—C15B—C16B	−120.8 (2)
C23A—C24A—C25A—O5A	177.3 (2)	C6B—C1B—C15B—C16B	62.7 (3)
O4A—C24A—C25A—C26A	178.4 (2)	O1B—C15B—C16B—C17B	61.8 (3)
C23A—C24A—C25A—C26A	−3.0 (3)	C14B—C15B—C16B—C17B	−56.2 (3)
O5A—C25A—C26A—C27A	−177.9 (2)	C1B—C15B—C16B—C17B	−177.87 (19)
C24A—C25A—C26A—C27A	2.4 (3)	C15B—C16B—C17B—C18B	−156.6 (2)
C23A—C22A—C27A—C26A	−1.8 (3)	C20B—N1B—C18B—C17B	69.5 (2)
C21A—C22A—C27A—C26A	177.7 (2)	C19B—N1B—C18B—C17B	−166.07 (19)
C25A—C26A—C27A—C22A	0.0 (3)	C16B—C17B—C18B—N1B	167.64 (19)

### \ (3-{2-Hydroxytricyclo[9.4.0.03,8]pentadeca-1(11),3,5,7,12,14-hexaen-\ 2-yl}propyl)dimethylazanium 3,4-dimethoxybenzoate trihydrate (II) Hydrogen-bond geometry (Å, º)

**Table:**

*D*—H···*A*	*D*—H	H···*A*	*D*···*A*	*D*—H···*A*
O1*A*—H1*OA*···O5*W*	0.82 (4)	1.95 (4)	2.762 (3)	172 (4)
N1*A*—H1*NA*···O2*A*	0.99 (3)	1.71 (3)	2.690 (3)	172 (3)
N1*A*—H1*NA*···O3*A*	0.99 (3)	2.45 (3)	3.108 (3)	124 (2)
C16*A*—H16*B*···O5*W*	0.99	2.61	3.308 (3)	128
C23*A*—H23*A*···O2*W*^i^	0.95	2.61	3.519 (3)	159
O1*B*—H1*OB*···O6*W*	0.87 (4)	1.91 (4)	2.781 (3)	173 (3)
N1*B*—H1*NB*···O2*B*	0.99 (3)	1.75 (3)	2.723 (3)	165 (2)
N1*B*—H1*NB*···O3*B*	0.99 (3)	2.48 (3)	3.186 (3)	128 (2)
C16*B*—H16*D*···O6*W*	0.99	2.46	3.134 (3)	125
O1*W*—H1*W*1···O3*A*	0.82 (2)	1.96 (2)	2.770 (3)	172 (4)
O1*W*—H2*W*1···O3*W*	0.81 (2)	2.02 (2)	2.795 (3)	161 (4)
O2*W*—H1*W*2···O2*A*^ii^	0.83 (2)	1.95 (2)	2.773 (3)	170 (4)
O2*W*—H2*W*2···O1*W*	0.83 (2)	1.93 (2)	2.738 (3)	166 (4)
O3*W*—H1*W*3···O3*B*^i^	0.83 (2)	1.96 (2)	2.785 (3)	178 (5)
O3*W*—H2*W*3···O4*W*	0.83 (2)	1.88 (2)	2.705 (4)	174 (5)
O4*W*—H1*W*4···O2*B*	0.82 (2)	1.89 (2)	2.708 (3)	171 (4)
O4*W*—H2*W*4···O2*W*	0.81 (2)	1.99 (2)	2.771 (3)	160 (4)
O5*W*—H1*W*5···O4*B*^iii^	0.83 (2)	2.36 (3)	3.035 (3)	138 (3)
O5*W*—H1*W*5···O5*B*^iii^	0.83 (2)	2.23 (2)	2.971 (2)	149 (3)
O5*W*—H2*W*5···O3*A*	0.83 (2)	2.00 (2)	2.820 (3)	173 (4)
O6*W*—H1*W*6···O4*A*^iv^	0.84 (2)	2.24 (3)	2.886 (2)	134 (3)
O6*W*—H1*W*6···O5*A*^iv^	0.84 (2)	2.23 (2)	2.980 (3)	150 (3)
O6*W*—H2*W*6···O3*B*	0.83 (2)	1.95 (2)	2.769 (3)	169 (3)

Symmetry codes: (i) *x*+1, *y*, *z*; (ii) *x*−1, *y*, *z*; (iii) *x*, *y*, *z*−1; (iv) *x*−1, *y*, *z*+1.

### (3-{2-Hydroxytricyclo[9.4.0.03,8]pentadeca-1(11),3,5,7,12,14-hexaen-2-yl}propyl)dimethylazanium 2-chlorobenzoate (III) Crystal data

**Table:**

C_20_H_26_NO^+^·C_7_H_4_ClO_2_^−^	*F*(000) = 960
*M**_r_* = 451.97	*D*_x_ = 1.303 Mg m^−^^3^
Monoclinic, *P*2_1_/*n*	Mo *K*α radiation, λ = 0.71073 Å
*a* = 6.7576 (2) Å	Cell parameters from 9897 reflections
*b* = 22.9081 (6) Å	θ = 2.9–27.5°
*c* = 14.9477 (3) Å	µ = 0.20 mm^−^^1^
β = 95.359 (1)°	*T* = 180 K
*V* = 2303.85 (10) Å^3^	Block, colourless
*Z* = 4	0.22 × 0.16 × 0.12 mm

### (3-{2-Hydroxytricyclo[9.4.0.03,8]pentadeca-1(11),3,5,7,12,14-hexaen-2-yl}propyl)dimethylazanium 2-chlorobenzoate (III) Data collection

**Table:**

Bruker D8 Venture dual source diffractometer	5279 independent reflections
Radiation source: microsource	4188 reflections with *I* > 2σ(*I*)
Detector resolution: 7.41 pixels mm^-1^	*R*_int_ = 0.039
φ and ω scans	θ_max_ = 27.5°, θ_min_ = 2.2°
Absorption correction: multi-scan (*SADABS*; Krause *et al.*, 2015)	*h* = −8→8
*T*_min_ = 0.848, *T*_max_ = 0.959	*k* = −29→29
35872 measured reflections	*l* = −19→19

### (3-{2-Hydroxytricyclo[9.4.0.03,8]pentadeca-1(11),3,5,7,12,14-hexaen-2-yl}propyl)dimethylazanium 2-chlorobenzoate (III) Refinement

**Table:**

Refinement on *F*^2^	Secondary atom site location: difference Fourier map
Least-squares matrix: full	Hydrogen site location: mixed
*R*[*F*^2^ > 2σ(*F*^2^)] = 0.045	H atoms treated by a mixture of independent and constrained refinement
*wR*(*F*^2^) = 0.110	*w* = 1/[σ^2^(*F*_o_^2^) + (0.0344*P*)^2^ + 1.4657*P*] where *P* = (*F*_o_^2^ + 2*F*_c_^2^)/3
*S* = 1.03	(Δ/σ)_max_ = 0.001
5279 reflections	Δρ_max_ = 0.63 e Å^−^^3^
380 parameters	Δρ_min_ = −0.61 e Å^−^^3^
404 restraints	Extinction correction: *SHELXL2019/2* (Sheldrick, 2015b), Fc^*^=kFc[1+0.001xFc^2^λ^3^/sin(2θ)]^-1/4^
Primary atom site location: structure-invariant direct methods	Extinction coefficient: 0.0055 (11)

### (3-{2-Hydroxytricyclo[9.4.0.03,8]pentadeca-1(11),3,5,7,12,14-hexaen-2-yl}propyl)dimethylazanium 2-chlorobenzoate (III) Special details

**Table:**

Experimental. The crystal was mounted using polyisobutene oil on the tip of a fine glass fibre, which was fastened in a copper mounting pin with electrical solder. It was placed directly into the cold gas stream of a liquid-nitrogen based cryostat (Hope, 1994; Parkin & Hope, 1998). The crystals appeared to undergo a destructive phase transition when cooled to 90K. Visual inspection of crystal integrity and diffraction quality vs temperature established a safe temperature for data collection of -93° C.
Geometry. All esds (except the esd in the dihedral angle between two l.s. planes) are estimated using the full covariance matrix. The cell esds are taken into account individually in the estimation of esds in distances, angles and torsion angles; correlations between esds in cell parameters are only used when they are defined by crystal symmetry. An approximate (isotropic) treatment of cell esds is used for estimating esds involving l.s. planes.
Refinement. Refinement progress was checked using *Platon* (Spek, 2020) and by an *R*-tensor (Parkin, 2000). The final model was further checked with the IUCr utility *checkCIF*.

### (3-{2-Hydroxytricyclo[9.4.0.03,8]pentadeca-1(11),3,5,7,12,14-hexaen-2-yl}propyl)dimethylazanium 2-chlorobenzoate (III) Fractional atomic coordinates and isotropic or equivalent isotropic displacement parameters (Å^2^)

**Table:**

	*x*	*y*	*z*	*U*_iso_*/*U*_eq_	Occ. (<1)
O1	1.08766 (18)	0.71374 (6)	0.68005 (9)	0.0388 (3)	
H1O	1.105 (3)	0.6787 (11)	0.6624 (15)	0.054 (7)*	
N1	0.6010 (2)	0.61029 (6)	0.46779 (9)	0.0297 (3)	
H1N	0.560 (3)	0.5931 (9)	0.5248 (14)	0.048 (6)*	
C1	0.8566 (3)	0.79026 (7)	0.68891 (11)	0.0340 (4)	
C2	1.0026 (3)	0.82305 (8)	0.65383 (12)	0.0452 (5)	
H2	1.119208	0.804054	0.637893	0.054*	
C3	0.9839 (4)	0.88324 (9)	0.64107 (14)	0.0583 (6)	
H3	1.087910	0.904653	0.617711	0.070*	
C4	0.8163 (4)	0.91134 (9)	0.66215 (13)	0.0519 (6)	
H4	0.802576	0.952324	0.654118	0.062*	
C5	0.6703 (3)	0.87968 (9)	0.69468 (15)	0.0523 (5)	
H5	0.552625	0.899131	0.708215	0.063*	
C6	0.6852 (3)	0.81968 (8)	0.70920 (17)	0.0521 (5)	
C7	0.5037 (4)	0.78841 (14)	0.7261 (2)	0.0413 (6)	0.585 (3)
H7A	0.479064	0.755636	0.683309	0.050*	0.585 (3)
H7B	0.387589	0.814958	0.719918	0.050*	0.585 (3)
C8	0.5401 (5)	0.76585 (19)	0.8220 (2)	0.0460 (7)	0.585 (3)
H8A	0.413604	0.748711	0.837966	0.055*	0.585 (3)
H8B	0.567508	0.800205	0.861352	0.055*	0.585 (3)
C9	0.7129 (3)	0.71860 (11)	0.84866 (13)	0.0536 (6)	
C7'	0.5369 (7)	0.7976 (2)	0.7851 (4)	0.0413 (6)	0.415 (3)
H7'A	0.584189	0.816270	0.842941	0.050*	0.415 (3)
H7'B	0.402748	0.813447	0.767179	0.050*	0.415 (3)
C8'	0.5133 (6)	0.7327 (2)	0.8032 (4)	0.0460 (7)	0.415 (3)
H8'A	0.406685	0.725218	0.842848	0.055*	0.415 (3)
H8'B	0.485756	0.710332	0.746685	0.055*	0.415 (3)
C10	0.7166 (4)	0.70071 (12)	0.93794 (14)	0.0628 (7)	
H10	0.604666	0.708795	0.969898	0.075*	
C11	0.8748 (4)	0.67204 (10)	0.98133 (14)	0.0553 (6)	
H11	0.872768	0.660390	1.042233	0.066*	
C12	1.0359 (4)	0.66044 (9)	0.93555 (14)	0.0543 (6)	
H12	1.148164	0.640996	0.964731	0.065*	
C13	1.0353 (3)	0.67713 (8)	0.84621 (13)	0.0427 (4)	
H13	1.147668	0.668280	0.814937	0.051*	
C14	0.8757 (2)	0.70636 (7)	0.80106 (11)	0.0328 (4)	
C15	0.8907 (2)	0.72385 (7)	0.70189 (11)	0.0306 (4)	
C16	0.7494 (2)	0.68667 (7)	0.63750 (11)	0.0302 (3)	
H16A	0.781845	0.644864	0.647117	0.036*	
H16B	0.610943	0.692780	0.652065	0.036*	
C17	0.7636 (3)	0.70177 (8)	0.53891 (11)	0.0376 (4)	
H17A	0.758925	0.744767	0.532353	0.045*	
H17B	0.894424	0.688522	0.521902	0.045*	
C18	0.6024 (3)	0.67544 (7)	0.47353 (11)	0.0373 (4)	
H18A	0.471694	0.688457	0.490970	0.045*	
H18B	0.617271	0.691320	0.412923	0.045*	
C19	0.7946 (3)	0.58483 (9)	0.44969 (13)	0.0428 (4)	
H19A	0.782727	0.542242	0.446047	0.064*	
H19B	0.895551	0.595334	0.498382	0.064*	
H19C	0.833659	0.600058	0.392648	0.064*	
C20	0.4418 (3)	0.59129 (9)	0.39854 (13)	0.0467 (5)	
H20A	0.466060	0.608124	0.340281	0.070*	
H20B	0.312640	0.604628	0.415442	0.070*	
H20C	0.441801	0.548605	0.394234	0.070*	
Cl1	−0.05707 (7)	0.52215 (2)	0.69053 (3)	0.04297 (16)	0.9600 (15)
O2	0.4875 (4)	0.55670 (15)	0.60797 (16)	0.0359 (6)	0.9600 (15)
O3	0.2218 (4)	0.61482 (13)	0.60465 (13)	0.0406 (4)	0.9600 (15)
C21	0.3407 (3)	0.57905 (8)	0.64190 (12)	0.0294 (4)	0.9600 (15)
C22	0.3192 (3)	0.56105 (7)	0.73778 (11)	0.0303 (4)	0.9600 (15)
C23	0.1495 (3)	0.53554 (7)	0.76610 (12)	0.0325 (4)	0.9600 (15)
C24	0.1385 (3)	0.52015 (9)	0.85523 (13)	0.0425 (5)	0.9600 (15)
H24	0.022568	0.501828	0.873292	0.051*	0.9600 (15)
C25	0.2976 (4)	0.53169 (9)	0.91742 (13)	0.0499 (5)	0.9600 (15)
H25	0.290500	0.521547	0.978665	0.060*	0.9600 (15)
C26	0.4667 (4)	0.55782 (9)	0.89141 (14)	0.0491 (5)	0.9600 (15)
H26	0.575063	0.566423	0.934541	0.059*	0.9600 (15)
C27	0.4770 (4)	0.57139 (12)	0.80175 (17)	0.0417 (5)	0.9600 (15)
H27	0.595325	0.588246	0.783629	0.050*	0.9600 (15)
Cl1'	0.564 (2)	0.5767 (8)	0.8173 (12)	0.053 (4)	0.0400 (15)
O2'	0.488 (9)	0.561 (4)	0.622 (5)	0.0359 (6)	0.0400 (15)
O3'	0.225 (10)	0.618 (3)	0.586 (4)	0.0406 (4)	0.0400 (15)
C21'	0.317 (5)	0.5805 (11)	0.6330 (19)	0.0294 (4)	0.0400 (15)
C22'	0.226 (3)	0.5557 (6)	0.7133 (13)	0.035 (3)	0.0400 (15)
C23'	0.322 (2)	0.5517 (6)	0.7991 (13)	0.042 (2)	0.0400 (15)
C24'	0.230 (3)	0.5285 (10)	0.8706 (14)	0.042 (3)	0.0400 (15)
H24'	0.299591	0.526275	0.928789	0.051*	0.0400 (15)
C25'	0.037 (4)	0.5087 (11)	0.8556 (17)	0.041 (3)	0.0400 (15)
H25'	−0.026900	0.492706	0.903833	0.049*	0.0400 (15)
C26'	−0.063 (3)	0.5119 (11)	0.7711 (18)	0.039 (3)	0.0400 (15)
H26'	−0.198774	0.501143	0.761498	0.047*	0.0400 (15)
C27'	0.039 (4)	0.531 (2)	0.7004 (16)	0.040 (3)	0.0400 (15)
H27'	−0.022587	0.527469	0.640815	0.048*	0.0400 (15)

### (3-{2-Hydroxytricyclo[9.4.0.03,8]pentadeca-1(11),3,5,7,12,14-hexaen-2-yl}propyl)dimethylazanium 2-chlorobenzoate (III) Atomic displacement parameters (Å^2^)

**Table:**

	*U*^11^	*U*^22^	*U*^33^	*U*^12^	*U*^13^	*U*^23^
O1	0.0272 (6)	0.0374 (7)	0.0526 (8)	−0.0017 (5)	0.0073 (5)	−0.0064 (6)
N1	0.0364 (7)	0.0252 (7)	0.0272 (7)	−0.0008 (6)	0.0006 (6)	−0.0023 (5)
C1	0.0378 (9)	0.0294 (8)	0.0326 (8)	−0.0031 (7)	−0.0077 (7)	−0.0076 (7)
C2	0.0652 (13)	0.0391 (10)	0.0330 (9)	−0.0016 (9)	0.0144 (9)	−0.0006 (8)
C3	0.0934 (18)	0.0407 (11)	0.0437 (11)	−0.0089 (12)	0.0217 (11)	0.0096 (9)
C4	0.0881 (17)	0.0306 (10)	0.0352 (10)	0.0043 (10)	−0.0035 (10)	−0.0006 (8)
C5	0.0570 (13)	0.0329 (10)	0.0637 (13)	0.0093 (9)	−0.0126 (10)	−0.0106 (9)
C6	0.0381 (10)	0.0302 (9)	0.0863 (16)	−0.0002 (8)	−0.004 (1)	−0.0095 (10)
C7	0.0304 (12)	0.0422 (14)	0.0501 (15)	0.0058 (10)	−0.0025 (12)	−0.0013 (13)
C8	0.0282 (12)	0.063 (2)	0.0472 (16)	0.0013 (16)	0.0083 (11)	−0.0021 (16)
C9	0.0333 (10)	0.0894 (16)	0.0375 (10)	0.003 (1)	0.0004 (8)	−0.0034 (10)
C7'	0.0304 (12)	0.0422 (14)	0.0501 (15)	0.0058 (10)	−0.0025 (12)	−0.0013 (13)
C8'	0.0282 (12)	0.063 (2)	0.0472 (16)	0.0013 (16)	0.0083 (11)	−0.0021 (16)
C10	0.0507 (13)	0.100 (2)	0.0389 (11)	0.0042 (13)	0.0082 (9)	−0.0066 (12)
C11	0.0745 (15)	0.0563 (13)	0.0349 (10)	0.0040 (11)	0.0031 (10)	−0.0071 (9)
C12	0.0716 (15)	0.0438 (11)	0.0456 (11)	0.0219 (10)	−0.0053 (10)	−0.0012 (9)
C13	0.0511 (11)	0.0340 (9)	0.043 (1)	0.0139 (8)	0.0038 (8)	−0.0046 (8)
C14	0.0331 (9)	0.0300 (8)	0.0345 (8)	−0.0054 (7)	−0.0013 (7)	−0.0097 (7)
C15	0.0251 (8)	0.0284 (8)	0.0377 (9)	−0.0008 (6)	0.0005 (6)	−0.0050 (7)
C16	0.0318 (8)	0.0249 (8)	0.0337 (8)	−0.0024 (6)	0.0021 (7)	−0.0041 (6)
C17	0.0509 (11)	0.0283 (8)	0.0335 (9)	−0.0088 (8)	0.0032 (8)	−0.0039 (7)
C18	0.0543 (11)	0.0248 (8)	0.0315 (8)	0.0009 (8)	−0.0031 (8)	−0.0010 (6)
C19	0.0442 (10)	0.042 (1)	0.0441 (10)	0.0038 (8)	0.0145 (8)	−0.0055 (8)
C20	0.0562 (12)	0.0408 (10)	0.0394 (10)	−0.0063 (9)	−0.0146 (9)	−0.0058 (8)
Cl1	0.0367 (3)	0.0428 (3)	0.0493 (3)	−0.0071 (2)	0.0036 (2)	0.0053 (2)
O2	0.0367 (7)	0.0329 (9)	0.0401 (12)	0.0059 (6)	0.0139 (7)	0.0008 (10)
O3	0.0401 (7)	0.0446 (8)	0.0377 (12)	0.0127 (6)	0.0075 (8)	0.0074 (8)
C21	0.0290 (9)	0.0262 (8)	0.0333 (8)	−0.0014 (6)	0.0047 (7)	−0.0028 (6)
C22	0.0326 (9)	0.0264 (8)	0.0323 (9)	0.0067 (7)	0.0052 (7)	−0.0017 (7)
C23	0.0391 (9)	0.0258 (8)	0.0334 (8)	0.0049 (7)	0.0070 (7)	−0.0007 (7)
C24	0.0569 (12)	0.0332 (10)	0.0398 (10)	0.0022 (9)	0.0167 (9)	0.0022 (8)
C25	0.0778 (15)	0.0420 (11)	0.0302 (9)	0.0102 (10)	0.0059 (10)	0.0026 (8)
C26	0.0595 (13)	0.0473 (12)	0.0382 (10)	0.0089 (10)	−0.0078 (9)	−0.0039 (9)
C27	0.0390 (13)	0.0429 (12)	0.0423 (12)	0.0051 (11)	−0.0003 (11)	−0.0012 (9)
Cl1'	0.059 (8)	0.051 (7)	0.050 (7)	0.004 (7)	0.006 (7)	0.003 (6)
O2'	0.0367 (7)	0.0329 (9)	0.0401 (12)	0.0059 (6)	0.0139 (7)	0.0008 (10)
O3'	0.0401 (7)	0.0446 (8)	0.0377 (12)	0.0127 (6)	0.0075 (8)	0.0074 (8)
C21'	0.0290 (9)	0.0262 (8)	0.0333 (8)	−0.0014 (6)	0.0047 (7)	−0.0028 (6)
C22'	0.038 (4)	0.030 (4)	0.038 (4)	0.004 (4)	0.005 (4)	−0.003 (4)
C23'	0.051 (4)	0.037 (4)	0.038 (4)	0.005 (4)	0.004 (4)	0.000 (4)
C24'	0.056 (4)	0.037 (4)	0.035 (4)	0.005 (4)	0.006 (4)	0.002 (4)
C25'	0.056 (5)	0.033 (5)	0.034 (5)	0.004 (5)	0.009 (5)	0.003 (5)
C26'	0.049 (5)	0.033 (5)	0.036 (5)	0.002 (5)	0.010 (5)	0.002 (5)
C27'	0.043 (6)	0.035 (6)	0.042 (6)	0.003 (6)	0.006 (6)	−0.001 (6)

### (3-{2-Hydroxytricyclo[9.4.0.03,8]pentadeca-1(11),3,5,7,12,14-hexaen-2-yl}propyl)dimethylazanium 2-chlorobenzoate (III) Geometric parameters (Å, º)

**Table:**

O1—C15	1.418 (2)	C16—C17	1.525 (2)
O1—H1O	0.86 (2)	C16—H16A	0.9900
N1—C19	1.480 (2)	C16—H16B	0.9900
N1—C20	1.487 (2)	C17—C18	1.519 (2)
N1—C18	1.495 (2)	C17—H17A	0.9900
N1—H1N	1.00 (2)	C17—H17B	0.9900
C1—C2	1.382 (3)	C18—H18A	0.9900
C1—C6	1.398 (3)	C18—H18B	0.9900
C1—C15	1.548 (2)	C19—H19A	0.9800
C2—C3	1.396 (3)	C19—H19B	0.9800
C2—H2	0.9500	C19—H19C	0.9800
C3—C4	1.365 (3)	C20—H20A	0.9800
C3—H3	0.9500	C20—H20B	0.9800
C4—C5	1.351 (3)	C20—H20C	0.9800
C4—H4	0.9500	Cl1—C23	1.7388 (19)
C5—C6	1.394 (3)	O2—C21	1.264 (2)
C5—H5	0.9500	O3—C21	1.243 (2)
C6—C7	1.463 (4)	C21—C22	1.511 (2)
C6—C7'	1.662 (5)	C22—C27	1.384 (3)
C7—C8	1.522 (4)	C22—C23	1.388 (2)
C7—H7A	0.9900	C23—C24	1.387 (3)
C7—H7B	0.9900	C24—C25	1.380 (3)
C8—C9	1.615 (4)	C24—H24	0.9500
C8—H8A	0.9900	C25—C26	1.378 (3)
C8—H8B	0.9900	C25—H25	0.9500
C9—C14	1.394 (3)	C26—C27	1.384 (3)
C9—C10	1.394 (3)	C26—H26	0.9500
C9—C8'	1.488 (5)	C27—H27	0.9500
C7'—C8'	1.523 (6)	Cl1'—C23'	1.729 (9)
C7'—H7'A	0.9900	O2'—C21'	1.264 (9)
C7'—H7'B	0.9900	O3'—C21'	1.243 (9)
C8'—H8'A	0.9900	C21'—C22'	1.510 (9)
C8'—H8'B	0.9900	C22'—C27'	1.38 (1)
C10—C11	1.366 (3)	C22'—C23'	1.385 (9)
C10—H10	0.9500	C23'—C24'	1.389 (9)
C11—C12	1.365 (3)	C24'—C25'	1.379 (10)
C11—H11	0.9500	C24'—H24'	0.9500
C12—C13	1.389 (3)	C25'—C26'	1.378 (10)
C12—H12	0.9500	C25'—H25'	0.9500
C13—C14	1.389 (2)	C26'—C27'	1.385 (10)
C13—H13	0.9500	C26'—H26'	0.9500
C14—C15	1.548 (2)	C27'—H27'	0.9500
C15—C16	1.547 (2)		
			
C15—O1—H1O	112.3 (15)	C16—C15—C1	112.76 (13)
C19—N1—C20	110.60 (14)	C14—C15—C1	110.61 (13)
C19—N1—C18	113.77 (14)	C17—C16—C15	112.66 (14)
C20—N1—C18	109.41 (13)	C17—C16—H16A	109.1
C19—N1—H1N	108.8 (12)	C15—C16—H16A	109.1
C20—N1—H1N	103.6 (12)	C17—C16—H16B	109.1
C18—N1—H1N	110.2 (12)	C15—C16—H16B	109.1
C2—C1—C6	117.12 (17)	H16A—C16—H16B	107.8
C2—C1—C15	118.58 (16)	C18—C17—C16	115.17 (15)
C6—C1—C15	124.29 (16)	C18—C17—H17A	108.5
C1—C2—C3	122.0 (2)	C16—C17—H17A	108.5
C1—C2—H2	119.0	C18—C17—H17B	108.5
C3—C2—H2	119.0	C16—C17—H17B	108.5
C4—C3—C2	120.0 (2)	H17A—C17—H17B	107.5
C4—C3—H3	120.0	N1—C18—C17	115.70 (14)
C2—C3—H3	120.0	N1—C18—H18A	108.4
C5—C4—C3	118.64 (19)	C17—C18—H18A	108.4
C5—C4—H4	120.7	N1—C18—H18B	108.4
C3—C4—H4	120.7	C17—C18—H18B	108.4
C4—C5—C6	122.8 (2)	H18A—C18—H18B	107.4
C4—C5—H5	118.6	N1—C19—H19A	109.5
C6—C5—H5	118.6	N1—C19—H19B	109.5
C5—C6—C1	119.4 (2)	H19A—C19—H19B	109.5
C5—C6—C7	117.4 (2)	N1—C19—H19C	109.5
C1—C6—C7	121.8 (2)	H19A—C19—H19C	109.5
C5—C6—C7'	111.6 (2)	H19B—C19—H19C	109.5
C1—C6—C7'	124.5 (2)	N1—C20—H20A	109.5
C6—C7—C8	105.3 (3)	N1—C20—H20B	109.5
C6—C7—H7A	110.7	H20A—C20—H20B	109.5
C8—C7—H7A	110.7	N1—C20—H20C	109.5
C6—C7—H7B	110.7	H20A—C20—H20C	109.5
C8—C7—H7B	110.7	H20B—C20—H20C	109.5
H7A—C7—H7B	108.8	O3—C21—O2	125.90 (17)
C7—C8—C9	120.7 (3)	O3—C21—C22	119.30 (15)
C7—C8—H8A	107.2	O2—C21—C22	114.76 (15)
C9—C8—H8A	107.2	C27—C22—C23	117.80 (18)
C7—C8—H8B	107.2	C27—C22—C21	118.17 (18)
C9—C8—H8B	107.2	C23—C22—C21	124.02 (16)
H8A—C8—H8B	106.8	C24—C23—C22	121.31 (18)
C14—C9—C10	119.00 (19)	C24—C23—Cl1	117.88 (15)
C14—C9—C8'	122.4 (3)	C22—C23—Cl1	120.80 (13)
C10—C9—C8'	115.9 (3)	C25—C24—C23	119.4 (2)
C14—C9—C8	126.6 (2)	C25—C24—H24	120.3
C10—C9—C8	112.5 (2)	C23—C24—H24	120.3
C8'—C7'—C6	119.9 (4)	C26—C25—C24	120.51 (19)
C8'—C7'—H7'A	107.4	C26—C25—H25	119.7
C6—C7'—H7'A	107.4	C24—C25—H25	119.7
C8'—C7'—H7'B	107.4	C25—C26—C27	119.3 (2)
C6—C7'—H7'B	107.4	C25—C26—H26	120.4
H7'A—C7'—H7'B	106.9	C27—C26—H26	120.4
C9—C8'—C7'	100.8 (4)	C26—C27—C22	121.7 (2)
C9—C8'—H8'A	111.6	C26—C27—H27	119.2
C7'—C8'—H8'A	111.6	C22—C27—H27	119.2
C9—C8'—H8'B	111.6	O3'—C21'—O2'	125.6 (16)
C7'—C8'—H8'B	111.6	O3'—C21'—C22'	119.5 (15)
H8'A—C8'—H8'B	109.4	O2'—C21'—C22'	114.8 (14)
C11—C10—C9	122.7 (2)	C27'—C22'—C23'	116.5 (10)
C11—C10—H10	118.7	C27'—C22'—C21'	118.5 (13)
C9—C10—H10	118.7	C23'—C22'—C21'	124.8 (13)
C12—C11—C10	118.7 (2)	C22'—C23'—C24'	122.3 (11)
C12—C11—H11	120.6	C22'—C23'—Cl1'	118.6 (11)
C10—C11—H11	120.6	C24'—C23'—Cl1'	119.2 (11)
C11—C12—C13	119.8 (2)	C25'—C24'—C23'	118.9 (12)
C11—C12—H12	120.1	C25'—C24'—H24'	120.6
C13—C12—H12	120.1	C23'—C24'—H24'	120.6
C12—C13—C14	122.15 (19)	C26'—C25'—C24'	120.5 (12)
C12—C13—H13	118.9	C26'—C25'—H25'	119.8
C14—C13—H13	118.9	C24'—C25'—H25'	119.8
C13—C14—C9	117.59 (17)	C25'—C26'—C27'	118.7 (12)
C13—C14—C15	118.19 (15)	C25'—C26'—H26'	120.6
C9—C14—C15	124.22 (16)	C27'—C26'—H26'	120.6
O1—C15—C16	107.76 (13)	C22'—C27'—C26'	122.4 (13)
O1—C15—C14	108.96 (13)	C22'—C27'—H27'	118.8
C16—C15—C14	111.19 (13)	C26'—C27'—H27'	118.8
O1—C15—C1	105.28 (13)		
			
C6—C1—C2—C3	1.7 (3)	C2—C1—C15—O1	5.5 (2)
C15—C1—C2—C3	−179.25 (17)	C6—C1—C15—O1	−175.44 (17)
C1—C2—C3—C4	−1.0 (3)	C2—C1—C15—C16	−111.70 (17)
C2—C3—C4—C5	−0.4 (3)	C6—C1—C15—C16	67.3 (2)
C3—C4—C5—C6	1.2 (3)	C2—C1—C15—C14	123.10 (17)
C4—C5—C6—C1	−0.6 (3)	C6—C1—C15—C14	−57.9 (2)
C4—C5—C6—C7	−167.3 (2)	O1—C15—C16—C17	−59.35 (18)
C4—C5—C6—C7'	156.6 (3)	C14—C15—C16—C17	−178.70 (14)
C2—C1—C6—C5	−0.9 (3)	C1—C15—C16—C17	56.41 (19)
C15—C1—C6—C5	−179.91 (18)	C15—C16—C17—C18	−168.84 (14)
C2—C1—C6—C7	165.2 (2)	C19—N1—C18—C17	−53.2 (2)
C15—C1—C6—C7	−13.8 (3)	C20—N1—C18—C17	−177.48 (16)
C2—C1—C6—C7'	−154.9 (3)	C16—C17—C18—N1	−64.1 (2)
C15—C1—C6—C7'	26.1 (3)	O3—C21—C22—C27	−120.5 (3)
C5—C6—C7—C8	−115.1 (3)	O2—C21—C22—C27	57.2 (3)
C1—C6—C7—C8	78.5 (3)	O3—C21—C22—C23	58.5 (3)
C6—C7—C8—C9	−64.1 (4)	O2—C21—C22—C23	−123.9 (3)
C7—C8—C9—C14	19.6 (5)	C27—C22—C23—C24	−0.8 (3)
C7—C8—C9—C10	−176.6 (3)	C21—C22—C23—C24	−179.78 (17)
C5—C6—C7'—C8'	173.2 (3)	C27—C22—C23—Cl1	178.91 (16)
C1—C6—C7'—C8'	−31.1 (5)	C21—C22—C23—Cl1	0.0 (2)
C14—C9—C8'—C7'	−83.4 (4)	C22—C23—C24—C25	1.6 (3)
C10—C9—C8'—C7'	115.6 (3)	Cl1—C23—C24—C25	−178.16 (15)
C6—C7'—C8'—C9	69.8 (5)	C23—C24—C25—C26	−0.5 (3)
C14—C9—C10—C11	0.7 (4)	C24—C25—C26—C27	−1.2 (3)
C8'—C9—C10—C11	162.3 (3)	C25—C26—C27—C22	2.0 (4)
C8—C9—C10—C11	−164.4 (3)	C23—C22—C27—C26	−1.0 (3)
C9—C10—C11—C12	0.1 (4)	C21—C22—C27—C26	178.0 (2)
C10—C11—C12—C13	−0.8 (3)	O3'—C21'—C22'—C27'	55 (6)
C11—C12—C13—C14	0.9 (3)	O2'—C21'—C22'—C27'	−127 (7)
C12—C13—C14—C9	−0.2 (3)	O3'—C21'—C22'—C23'	−130 (6)
C12—C13—C14—C15	179.15 (18)	O2'—C21'—C22'—C23'	49 (7)
C10—C9—C14—C13	−0.6 (3)	C27'—C22'—C23'—C24'	−5 (3)
C8'—C9—C14—C13	−161.0 (3)	C21'—C22'—C23'—C24'	180.00 (17)
C8—C9—C14—C13	162.2 (2)	C27'—C22'—C23'—Cl1'	175 (2)
C10—C9—C14—C15	−179.9 (2)	C21'—C22'—C23'—Cl1'	0.0 (2)
C8'—C9—C14—C15	19.8 (4)	C22'—C23'—C24'—C25'	0.0 (4)
C8—C9—C14—C15	−17.1 (3)	Cl1'—C23'—C24'—C25'	180.0 (2)
C13—C14—C15—O1	−9.3 (2)	C23'—C24'—C25'—C26'	0.0 (4)
C9—C14—C15—O1	169.98 (17)	C24'—C25'—C26'—C27'	5 (3)
C13—C14—C15—C16	109.36 (17)	C23'—C22'—C27'—C26'	10 (5)
C9—C14—C15—C16	−71.4 (2)	C21'—C22'—C27'—C26'	−174 (3)
C13—C14—C15—C1	−124.55 (16)	C25'—C26'—C27'—C22'	−10 (5)
C9—C14—C15—C1	54.7 (2)		

### (3-{2-Hydroxytricyclo[9.4.0.03,8]pentadeca-1(11),3,5,7,12,14-hexaen-2-yl}propyl)dimethylazanium 2-chlorobenzoate (III) Hydrogen-bond geometry (Å, º)

**Table:**

*D*—H···*A*	*D*—H	H···*A*	*D*···*A*	*D*—H···*A*
O1—H1*O*···O3^i^	0.86 (2)	1.91 (2)	2.724 (3)	159 (2)
O1—H1*O*···O3′^i^	0.86 (2)	2.02 (8)	2.81 (8)	153 (3)
N1—H1*N*···O2	1.00 (2)	1.61 (2)	2.605 (2)	171.5 (19)
N1—H1*N*···O2′	1.00 (2)	1.73 (4)	2.73 (4)	178 (4)
N1—H1*N*···O3′	1.00 (2)	2.58 (5)	3.23 (5)	122.4 (16)
C19—H19*B*···O3^i^	0.98	2.63	3.595 (4)	167
C19—H19*B*···O3′^i^	0.98	2.52	3.47 (9)	162
C20—H20*C*···O2^ii^	0.98	2.46	3.426 (4)	169
C20—H20*C*···O2′^ii^	0.98	2.57	3.54 (10)	169

Symmetry codes: (i) *x*+1, *y*, *z*; (ii) −*x*+1, −*y*+1, −*z*+1.

### (3-{2-Hydroxytricyclo[9.4.0.03,8]pentadeca-1(11),3,5,7,12,14-hexaen-2-yl}propyl)dimethylazanium thiophene-2-carboxylate monohydrate (IV) Crystal data

**Table:**

C_20_H_26_NO^+^·C_5_H_3_O_2_S^−^·H_2_O	*D*_x_ = 1.308 Mg m^−^^3^
*M**_r_* = 441.57	Mo *K*α radiation, λ = 0.71073 Å
Orthorhombic, *P*2_1_2_1_2_1_	Cell parameters from 9921 reflections
*a* = 6.1659 (5) Å	θ = 2.7–27.5°
*b* = 13.1299 (12) Å	µ = 0.18 mm^−^^1^
*c* = 27.698 (2) Å	*T* = 90 K
*V* = 2242.3 (3) Å^3^	Tablet, colourless
*Z* = 4	0.27 × 0.13 × 0.04 mm
*F*(000) = 944	

### (3-{2-Hydroxytricyclo[9.4.0.03,8]pentadeca-1(11),3,5,7,12,14-hexaen-2-yl}propyl)dimethylazanium thiophene-2-carboxylate monohydrate (IV) Data collection

**Table:**

Bruker D8 Venture dual source diffractometer	5133 independent reflections
Radiation source: microsource	4772 reflections with *I* > 2σ(*I*)
Detector resolution: 7.41 pixels mm^-1^	*R*_int_ = 0.042
φ and ω scans	θ_max_ = 27.5°, θ_min_ = 2.1°
Absorption correction: multi-scan (*SADABS*; Krause *et al.*, 2015)	*h* = −8→7
*T*_min_ = 0.852, *T*_max_ = 0.959	*k* = −17→17
43123 measured reflections	*l* = −36→36

### (3-{2-Hydroxytricyclo[9.4.0.03,8]pentadeca-1(11),3,5,7,12,14-hexaen-2-yl}propyl)dimethylazanium thiophene-2-carboxylate monohydrate (IV) Refinement

**Table:**

Refinement on *F*^2^	Secondary atom site location: difference Fourier map
Least-squares matrix: full	Hydrogen site location: mixed
*R*[*F*^2^ > 2σ(*F*^2^)] = 0.028	H atoms treated by a mixture of independent and constrained refinement
*wR*(*F*^2^) = 0.062	*w* = 1/[σ^2^(*F*_o_^2^) + (0.0234*P*)^2^ + 0.459*P*] where *P* = (*F*_o_^2^ + 2*F*_c_^2^)/3
*S* = 1.06	(Δ/σ)_max_ = 0.001
5133 reflections	Δρ_max_ = 0.19 e Å^−^^3^
315 parameters	Δρ_min_ = −0.19 e Å^−^^3^
10 restraints	Absolute structure: Twinned by inversion.
Primary atom site location: structure-invariant direct methods	Absolute structure parameter: 0.30 (7)

### (3-{2-Hydroxytricyclo[9.4.0.03,8]pentadeca-1(11),3,5,7,12,14-hexaen-2-yl}propyl)dimethylazanium thiophene-2-carboxylate monohydrate (IV) Special details

**Table:**

Experimental. The crystal was mounted using polyisobutene oil on the tip of a fine glass fibre, which was fastened in a copper mounting pin with electrical solder. It was placed directly into the cold gas stream of a liquid-nitrogen based cryostat (Hope, 1994; Parkin & Hope, 1998). Diffraction data were collected with the crystal at 90K, which is standard practice in this laboratory for the majority of flash-cooled crystals.
Geometry. All esds (except the esd in the dihedral angle between two l.s. planes) are estimated using the full covariance matrix. The cell esds are taken into account individually in the estimation of esds in distances, angles and torsion angles; correlations between esds in cell parameters are only used when they are defined by crystal symmetry. An approximate (isotropic) treatment of cell esds is used for estimating esds involving l.s. planes.
Refinement. Refined as a 2-component inversion twin.

### (3-{2-Hydroxytricyclo[9.4.0.03,8]pentadeca-1(11),3,5,7,12,14-hexaen-2-yl}propyl)dimethylazanium thiophene-2-carboxylate monohydrate (IV) Fractional atomic coordinates and isotropic or equivalent isotropic displacement parameters (Å^2^)

**Table:**

	*x*	*y*	*z*	*U*_iso_*/*U*_eq_	Occ. (<1)
O1	0.0126 (2)	0.58778 (10)	0.63942 (4)	0.0166 (3)	
H1O	0.004 (4)	0.6031 (17)	0.6681 (8)	0.029 (6)*	
N1	0.4459 (3)	0.42400 (12)	0.73556 (5)	0.0181 (3)	
H1N	0.414 (3)	0.4876 (19)	0.7500 (7)	0.028 (6)*	
C1	0.1911 (3)	0.74788 (14)	0.61758 (6)	0.0145 (4)	
C2	0.0149 (3)	0.79475 (14)	0.64074 (6)	0.0181 (4)	
H2	−0.091994	0.753200	0.655643	0.022*	
C3	−0.0081 (3)	0.89955 (15)	0.64259 (6)	0.0215 (4)	
H3	−0.131587	0.928973	0.657624	0.026*	
C4	0.1494 (3)	0.96115 (15)	0.62245 (6)	0.0219 (4)	
H4	0.138751	1.033189	0.624603	0.026*	
C5	0.3224 (3)	0.91624 (15)	0.59916 (6)	0.0199 (4)	
H5	0.430753	0.958886	0.585524	0.024*	
C6	0.3454 (3)	0.81054 (14)	0.59471 (6)	0.0166 (4)	
C7	0.5327 (3)	0.77912 (15)	0.56226 (6)	0.0193 (4)	
H7A	0.661250	0.818537	0.572726	0.023*	
H7B	0.496813	0.802372	0.529210	0.023*	
C8	0.6028 (3)	0.66828 (15)	0.55839 (6)	0.0181 (4)	
H8A	0.723454	0.663333	0.534937	0.022*	
H8B	0.658328	0.645680	0.590169	0.022*	
C9	0.4243 (3)	0.59754 (14)	0.54289 (6)	0.0171 (4)	
C10	0.4468 (3)	0.54576 (15)	0.49902 (6)	0.0212 (4)	
H10	0.573560	0.556479	0.480189	0.025*	
C11	0.2895 (3)	0.47934 (15)	0.48231 (6)	0.0229 (4)	
H11	0.308467	0.444647	0.452511	0.027*	
C12	0.1043 (3)	0.46409 (15)	0.50952 (6)	0.0204 (4)	
H12	−0.005380	0.418995	0.498405	0.025*	
C13	0.0789 (3)	0.51493 (14)	0.55318 (6)	0.0170 (4)	
H13	−0.048939	0.504153	0.571617	0.020*	
C14	0.2371 (3)	0.58134 (14)	0.57047 (6)	0.0148 (4)	
C15	0.2083 (3)	0.63035 (14)	0.62048 (6)	0.0141 (3)	
C16	0.3924 (3)	0.59993 (14)	0.65624 (6)	0.0153 (4)	
H16A	0.518832	0.645177	0.651203	0.018*	
H16B	0.340931	0.609543	0.689787	0.018*	
C17	0.4623 (3)	0.48945 (14)	0.64929 (6)	0.0176 (4)	
H17A	0.331425	0.448170	0.642543	0.021*	
H17B	0.556508	0.485789	0.620391	0.021*	
C18	0.5823 (3)	0.44131 (15)	0.69130 (6)	0.0177 (4)	
H18A	0.642223	0.375076	0.680643	0.021*	
H18B	0.705992	0.485638	0.700092	0.021*	
C19	0.2363 (3)	0.37253 (17)	0.72515 (7)	0.0252 (4)	
H19A	0.143055	0.418364	0.706473	0.038*	
H19B	0.164428	0.354959	0.755588	0.038*	
H19C	0.263284	0.310346	0.706529	0.038*	
C20	0.5736 (4)	0.36513 (18)	0.77156 (7)	0.0337 (5)	
H20A	0.489898	0.358568	0.801467	0.051*	
H20B	0.709976	0.400774	0.778277	0.051*	
H20C	0.605215	0.297237	0.758641	0.051*	
O2	0.3116 (2)	0.58314 (11)	0.78720 (5)	0.0226 (3)	
O3	0.6468 (2)	0.63027 (12)	0.80797 (5)	0.0242 (3)	
C21	0.4471 (3)	0.62759 (14)	0.81440 (6)	0.0170 (4)	
C22	0.3557 (6)	0.6824 (7)	0.8572 (3)	0.0153 (5)	0.899 (3)
S1	0.53403 (11)	0.72881 (9)	0.89993 (2)	0.01932 (17)	0.899 (3)
C23	0.3261 (4)	0.7847 (2)	0.93133 (10)	0.0216 (6)	0.899 (3)
H23	0.345230	0.822272	0.960340	0.026*	0.899 (3)
C24	0.1299 (4)	0.7691 (4)	0.90976 (12)	0.0214 (6)	0.899 (3)
H24	−0.003283	0.795199	0.921778	0.026*	0.899 (3)
C25	0.1477 (15)	0.7099 (11)	0.8678 (4)	0.0232 (11)	0.899 (3)
H25	0.026638	0.690741	0.848600	0.028*	0.899 (3)
C22'	0.388 (5)	0.678 (7)	0.857 (2)	0.0153 (5)	0.101 (3)
S1'	0.118 (4)	0.707 (3)	0.8642 (10)	0.0232 (11)	0.101 (3)
C23'	0.171 (4)	0.770 (4)	0.9170 (13)	0.0214 (6)	0.101 (3)
H23'	0.062801	0.799212	0.937142	0.026*	0.101 (3)
C24'	0.387 (4)	0.775 (3)	0.9266 (10)	0.0216 (6)	0.101 (3)
H24'	0.447583	0.807427	0.954137	0.026*	0.101 (3)
C25'	0.511 (4)	0.726 (4)	0.8910 (12)	0.01932 (17)	0.101 (3)
H25'	0.664620	0.725448	0.890806	0.023*	0.101 (3)
O1W	0.9441 (3)	0.62270 (13)	0.73651 (5)	0.0304 (4)	
H1W	0.834 (5)	0.619 (2)	0.7559 (10)	0.058 (9)*	
H2W	1.053 (5)	0.614 (2)	0.7533 (11)	0.056 (9)*	

### (3-{2-Hydroxytricyclo[9.4.0.03,8]pentadeca-1(11),3,5,7,12,14-hexaen-2-yl}propyl)dimethylazanium thiophene-2-carboxylate monohydrate (IV) Atomic displacement parameters (Å^2^)

**Table:**

	*U*^11^	*U*^22^	*U*^33^	*U*^12^	*U*^13^	*U*^23^
O1	0.0163 (6)	0.0188 (7)	0.0148 (6)	−0.0031 (5)	0.0036 (5)	−0.0023 (5)
N1	0.0268 (8)	0.0129 (8)	0.0147 (7)	0.0034 (7)	−0.0032 (6)	−0.0009 (6)
C1	0.0163 (8)	0.0145 (10)	0.0128 (7)	0.0022 (7)	−0.0036 (6)	−0.0008 (6)
C2	0.0196 (9)	0.0187 (10)	0.0158 (8)	0.0019 (7)	−0.0009 (7)	−0.0015 (6)
C3	0.025 (1)	0.0214 (10)	0.0180 (8)	0.0085 (8)	−0.0037 (7)	−0.0040 (7)
C4	0.0352 (11)	0.0126 (10)	0.0178 (8)	0.0028 (8)	−0.0073 (8)	−0.0015 (7)
C5	0.0262 (9)	0.0163 (10)	0.0172 (8)	−0.0039 (8)	−0.0056 (8)	0.0017 (7)
C6	0.0189 (9)	0.0162 (9)	0.0147 (8)	0.0010 (7)	−0.0055 (7)	0.0001 (7)
C7	0.0175 (8)	0.0204 (10)	0.0198 (8)	−0.0030 (8)	−0.0001 (7)	0.0037 (7)
C8	0.0163 (9)	0.0211 (11)	0.0169 (8)	0.0004 (7)	0.0032 (7)	0.0019 (7)
C9	0.0192 (9)	0.0164 (10)	0.0158 (8)	0.0041 (7)	0.0012 (7)	0.0018 (7)
C10	0.0265 (10)	0.022 (1)	0.0150 (8)	0.0043 (9)	0.0038 (8)	0.0017 (7)
C11	0.0358 (11)	0.0184 (10)	0.0145 (8)	0.0037 (8)	0.0014 (8)	−0.0030 (7)
C12	0.0278 (10)	0.0157 (10)	0.0178 (8)	−0.0024 (8)	−0.0036 (7)	−0.0003 (7)
C13	0.0205 (9)	0.0142 (9)	0.0164 (8)	0.0007 (7)	0.0011 (7)	0.0018 (7)
C14	0.0179 (8)	0.0125 (9)	0.0140 (8)	0.0045 (7)	0.0007 (7)	0.0007 (6)
C15	0.0141 (8)	0.0148 (9)	0.0135 (7)	−0.0007 (7)	0.0012 (6)	0.0002 (7)
C16	0.0167 (8)	0.0138 (9)	0.0152 (8)	−0.0003 (7)	0.0009 (6)	0.0005 (7)
C17	0.0228 (9)	0.0156 (9)	0.0142 (7)	0.0028 (8)	0.0017 (7)	−0.0004 (7)
C18	0.0171 (9)	0.0152 (9)	0.0208 (8)	0.0029 (7)	−0.0006 (7)	−0.0009 (7)
C19	0.0296 (11)	0.0199 (11)	0.0262 (9)	−0.0055 (9)	0.0056 (8)	−0.0005 (8)
C20	0.0543 (15)	0.0287 (12)	0.0182 (9)	0.0143 (11)	−0.0105 (10)	0.0025 (8)
O2	0.0240 (7)	0.0234 (8)	0.0206 (6)	0.0040 (6)	−0.0017 (5)	−0.0063 (6)
O3	0.0198 (7)	0.0296 (8)	0.0230 (7)	0.0025 (6)	0.0058 (5)	0.0011 (6)
C21	0.0212 (9)	0.0131 (9)	0.0167 (8)	0.0030 (8)	0.0032 (7)	0.0034 (7)
C22	0.0175 (14)	0.0110 (14)	0.0175 (8)	−0.0007 (18)	0.0022 (14)	0.0020 (7)
S1	0.0196 (3)	0.0193 (3)	0.0191 (4)	−0.0007 (3)	−0.0014 (2)	−0.0019 (3)
C23	0.0241 (15)	0.0193 (13)	0.0213 (10)	−0.0003 (12)	0.0057 (11)	−0.0066 (8)
C24	0.0202 (13)	0.019 (1)	0.0252 (15)	0.0006 (14)	0.0037 (10)	−0.0035 (12)
C25	0.028 (3)	0.0164 (12)	0.0247 (19)	−0.002 (2)	0.0019 (17)	−0.0015 (12)
C22'	0.0175 (14)	0.0110 (14)	0.0175 (8)	−0.0007 (18)	0.0022 (14)	0.0020 (7)
S1'	0.028 (3)	0.0164 (12)	0.0247 (19)	−0.002 (2)	0.0019 (17)	−0.0015 (12)
C23'	0.0202 (13)	0.019 (1)	0.0252 (15)	0.0006 (14)	0.0037 (10)	−0.0035 (12)
C24'	0.0241 (15)	0.0193 (13)	0.0213 (10)	−0.0003 (12)	0.0057 (11)	−0.0066 (8)
C25'	0.0196 (3)	0.0193 (3)	0.0191 (4)	−0.0007 (3)	−0.0014 (2)	−0.0019 (3)
O1W	0.0197 (7)	0.0532 (11)	0.0182 (6)	0.0024 (8)	0.0029 (6)	−0.0039 (7)

### (3-{2-Hydroxytricyclo[9.4.0.03,8]pentadeca-1(11),3,5,7,12,14-hexaen-2-yl}propyl)dimethylazanium thiophene-2-carboxylate monohydrate (IV) Geometric parameters (Å, º)

**Table:**

O1—C15	1.430 (2)	C16—C17	1.525 (2)
O1—H1O	0.82 (2)	C16—H16A	0.9900
N1—C19	1.487 (3)	C16—H16B	0.9900
N1—C20	1.487 (2)	C17—C18	1.517 (2)
N1—C18	1.504 (2)	C17—H17A	0.9900
N1—H1N	0.95 (2)	C17—H17B	0.9900
C1—C2	1.404 (2)	C18—H18A	0.9900
C1—C6	1.408 (3)	C18—H18B	0.9900
C1—C15	1.549 (2)	C19—H19A	0.9800
C2—C3	1.384 (3)	C19—H19B	0.9800
C2—H2	0.9500	C19—H19C	0.9800
C3—C4	1.382 (3)	C20—H20A	0.9800
C3—H3	0.9500	C20—H20B	0.9800
C4—C5	1.379 (3)	C20—H20C	0.9800
C4—H4	0.9500	O2—C21	1.267 (2)
C5—C6	1.401 (3)	O3—C21	1.245 (2)
C5—H5	0.9500	C21—C22'	1.40 (3)
C6—C7	1.521 (3)	C21—C22	1.496 (3)
C7—C8	1.522 (3)	C22—C25	1.364 (9)
C7—H7A	0.9900	C22—S1	1.727 (3)
C7—H7B	0.9900	S1—C23	1.714 (2)
C8—C9	1.502 (3)	C23—C24	1.365 (3)
C8—H8A	0.9900	C23—H23	0.9500
C8—H8B	0.9900	C24—C25	1.403 (10)
C9—C10	1.399 (2)	C24—H24	0.9500
C9—C14	1.401 (2)	C25—H25	0.9500
C10—C11	1.384 (3)	C22'—C25'	1.367 (16)
C10—H10	0.9500	C22'—S1'	1.720 (14)
C11—C12	1.383 (3)	S1'—C23'	1.716 (13)
C11—H11	0.9500	C23'—C24'	1.359 (13)
C12—C13	1.390 (2)	C23'—H23'	0.9500
C12—H12	0.9500	C24'—C25'	1.402 (16)
C13—C14	1.393 (3)	C24'—H24'	0.9500
C13—H13	0.9500	C25'—H25'	0.9500
C14—C15	1.538 (2)	O1W—H1W	0.87 (3)
C15—C16	1.559 (2)	O1W—H2W	0.83 (3)
			
C15—O1—H1O	108.3 (17)	C17—C16—H16A	109.3
C19—N1—C20	110.74 (17)	C15—C16—H16A	109.3
C19—N1—C18	113.36 (14)	C17—C16—H16B	109.3
C20—N1—C18	109.20 (15)	C15—C16—H16B	109.3
C19—N1—H1N	107.6 (13)	H16A—C16—H16B	107.9
C20—N1—H1N	106.5 (13)	C18—C17—C16	115.93 (14)
C18—N1—H1N	109.1 (13)	C18—C17—H17A	108.3
C2—C1—C6	118.18 (17)	C16—C17—H17A	108.3
C2—C1—C15	117.77 (16)	C18—C17—H17B	108.3
C6—C1—C15	124.00 (15)	C16—C17—H17B	108.3
C3—C2—C1	122.14 (17)	H17A—C17—H17B	107.4
C3—C2—H2	118.9	N1—C18—C17	114.57 (15)
C1—C2—H2	118.9	N1—C18—H18A	108.6
C4—C3—C2	119.67 (18)	C17—C18—H18A	108.6
C4—C3—H3	120.2	N1—C18—H18B	108.6
C2—C3—H3	120.2	C17—C18—H18B	108.6
C5—C4—C3	118.84 (19)	H18A—C18—H18B	107.6
C5—C4—H4	120.6	N1—C19—H19A	109.5
C3—C4—H4	120.6	N1—C19—H19B	109.5
C4—C5—C6	122.90 (18)	H19A—C19—H19B	109.5
C4—C5—H5	118.5	N1—C19—H19C	109.5
C6—C5—H5	118.5	H19A—C19—H19C	109.5
C5—C6—C1	118.10 (17)	H19B—C19—H19C	109.5
C5—C6—C7	113.43 (16)	N1—C20—H20A	109.5
C1—C6—C7	128.36 (17)	N1—C20—H20B	109.5
C6—C7—C8	121.10 (15)	H20A—C20—H20B	109.5
C6—C7—H7A	107.1	N1—C20—H20C	109.5
C8—C7—H7A	107.1	H20A—C20—H20C	109.5
C6—C7—H7B	107.1	H20B—C20—H20C	109.5
C8—C7—H7B	107.1	O3—C21—O2	125.44 (17)
H7A—C7—H7B	106.8	O3—C21—C22'	111.5 (15)
C9—C8—C7	113.79 (15)	O2—C21—C22'	123.0 (16)
C9—C8—H8A	108.8	O3—C21—C22	118.2 (2)
C7—C8—H8A	108.8	O2—C21—C22	116.4 (2)
C9—C8—H8B	108.8	C25—C22—C21	130.7 (5)
C7—C8—H8B	108.8	C25—C22—S1	111.0 (4)
H8A—C8—H8B	107.7	C21—C22—S1	118.2 (2)
C10—C9—C14	118.78 (18)	C23—S1—C22	91.31 (12)
C10—C9—C8	118.43 (17)	C24—C23—S1	112.10 (19)
C14—C9—C8	122.79 (15)	C24—C23—H23	123.9
C11—C10—C9	121.80 (18)	S1—C23—H23	123.9
C11—C10—H10	119.1	C23—C24—C25	112.1 (4)
C9—C10—H10	119.1	C23—C24—H24	123.9
C12—C11—C10	119.20 (17)	C25—C24—H24	123.9
C12—C11—H11	120.4	C22—C25—C24	113.5 (5)
C10—C11—H11	120.4	C22—C25—H25	123.3
C11—C12—C13	119.83 (18)	C24—C25—H25	123.3
C11—C12—H12	120.1	C25'—C22'—C21	131 (3)
C13—C12—H12	120.1	C25'—C22'—S1'	110.6 (12)
C12—C13—C14	121.39 (17)	C21—C22'—S1'	117 (2)
C12—C13—H13	119.3	C23'—S1'—C22'	91.3 (9)
C14—C13—H13	119.3	C24'—C23'—S1'	112.1 (13)
C13—C14—C9	118.99 (15)	C24'—C23'—H23'	123.9
C13—C14—C15	119.39 (15)	S1'—C23'—H23'	123.9
C9—C14—C15	121.54 (16)	C23'—C24'—C25'	111.9 (14)
O1—C15—C14	105.33 (14)	C23'—C24'—H24'	124.0
O1—C15—C1	110.54 (14)	C25'—C24'—H24'	124.0
C14—C15—C1	112.21 (14)	C22'—C25'—C24'	113.5 (15)
O1—C15—C16	106.35 (13)	C22'—C25'—H25'	123.2
C14—C15—C16	112.40 (14)	C24'—C25'—H25'	123.2
C1—C15—C16	109.76 (14)	H1W—O1W—H2W	106 (2)
C17—C16—C15	111.67 (14)		
			
C6—C1—C2—C3	−1.5 (2)	C6—C1—C15—O1	−171.35 (15)
C15—C1—C2—C3	176.23 (16)	C2—C1—C15—C14	128.37 (16)
C1—C2—C3—C4	−2.1 (3)	C6—C1—C15—C14	−54.1 (2)
C2—C3—C4—C5	2.6 (3)	C2—C1—C15—C16	−105.89 (17)
C3—C4—C5—C6	0.4 (3)	C6—C1—C15—C16	71.6 (2)
C4—C5—C6—C1	−3.8 (3)	O1—C15—C16—C17	77.64 (17)
C4—C5—C6—C7	172.66 (16)	C14—C15—C16—C17	−37.1 (2)
C2—C1—C6—C5	4.2 (2)	C1—C15—C16—C17	−162.76 (14)
C15—C1—C6—C5	−173.28 (15)	C15—C16—C17—C18	−160.52 (15)
C2—C1—C6—C7	−171.65 (16)	C19—N1—C18—C17	49.0 (2)
C15—C1—C6—C7	10.8 (3)	C20—N1—C18—C17	172.95 (16)
C5—C6—C7—C8	171.58 (16)	C16—C17—C18—N1	68.6 (2)
C1—C6—C7—C8	−12.4 (3)	O3—C21—C22—C25	164.9 (11)
C6—C7—C8—C9	56.9 (2)	O2—C21—C22—C25	−14.1 (14)
C7—C8—C9—C10	116.72 (18)	O3—C21—C22—S1	−9.9 (9)
C7—C8—C9—C14	−63.3 (2)	O2—C21—C22—S1	171.0 (5)
C14—C9—C10—C11	0.0 (3)	C25—C22—S1—C23	−0.2 (9)
C8—C9—C10—C11	180.00 (18)	C21—C22—S1—C23	175.7 (7)
C9—C10—C11—C12	0.4 (3)	C22—S1—C23—C24	−0.4 (5)
C10—C11—C12—C13	−0.3 (3)	S1—C23—C24—C25	0.9 (8)
C11—C12—C13—C14	−0.1 (3)	C21—C22—C25—C24	−174.5 (9)
C12—C13—C14—C9	0.6 (3)	S1—C22—C25—C24	0.7 (14)
C12—C13—C14—C15	−176.05 (16)	C23—C24—C25—C22	−1.0 (13)
C10—C9—C14—C13	−0.5 (3)	O3—C21—C22'—C25'	−2 (12)
C8—C9—C14—C13	179.54 (17)	O2—C21—C22'—C25'	177 (8)
C10—C9—C14—C15	176.04 (16)	O3—C21—C22'—S1'	165 (5)
C8—C9—C14—C15	−3.9 (3)	O2—C21—C22'—S1'	−16 (10)
C13—C14—C15—O1	2.8 (2)	C25'—C22'—S1'—C23'	−6 (7)
C9—C14—C15—O1	−173.68 (15)	C21—C22'—S1'—C23'	−176 (7)
C13—C14—C15—C1	−117.49 (18)	C22'—S1'—C23'—C24'	4 (6)
C9—C14—C15—C1	66.0 (2)	S1'—C23'—C24'—C25'	0 (6)
C13—C14—C15—C16	118.22 (18)	C21—C22'—C25'—C24'	175 (8)
C9—C14—C15—C16	−58.3 (2)	S1'—C22'—C25'—C24'	7 (9)
C2—C1—C15—O1	11.1 (2)	C23'—C24'—C25'—C22'	−5 (8)

### (3-{2-Hydroxytricyclo[9.4.0.03,8]pentadeca-1(11),3,5,7,12,14-hexaen-2-yl}propyl)dimethylazanium thiophene-2-carboxylate monohydrate (IV) Hydrogen-bond geometry (Å, º)

**Table:**

*D*—H···*A*	*D*—H	H···*A*	*D*···*A*	*D*—H···*A*
O1—H1*O*···O1*W*^i^	0.82 (2)	1.95 (2)	2.7607 (18)	170 (2)
N1—H1*N*···O2	0.95 (2)	1.74 (2)	2.664 (2)	164 (2)
C18—H18*A*···S1′b^ii^	0.99	2.93	3.90 (3)	167
C18—H18*B*···O1*W*	0.99	2.53	3.495 (3)	164
C19—H19*C*···O3^ii^	0.98	2.46	3.388 (3)	158
C25′b—H25′b···S1′b^iii^	0.95	2.90	3.82 (3)	165
O1*W*—H1*W*···O3	0.87 (3)	1.85 (3)	2.6993 (19)	165 (3)
O1*W*—H2*W*···O2^iii^	0.83 (3)	1.89 (3)	2.716 (2)	174 (3)

Symmetry codes: (i) *x*−1, *y*, *z*; (ii) −*x*+1, *y*−1/2, −*z*+3/2; (iii) *x*+1, *y*, *z*.
